# Effects of different rehabilitation strategies on physical function and complications in postoperative patients with esophageal cancer: a systematic review and meta-analysis

**DOI:** 10.3389/fpubh.2026.1788265

**Published:** 2026-04-14

**Authors:** Ting Shen, Zihan Yi, Min Chen, Xiaoqing Zhang, Mengling Gu, Lin Luo, Xiahong Huang

**Affiliations:** Deyang People’s Hospital, Deyang, China

**Keywords:** esophageal cancer, pulmonary complications, rehabilitation, postoperative care, physical therapy, respiratory function, multimodal intervention, clinical outcomes

## Abstract

**Background:**

Pulmonary complications remain a major challenge after esophagectomy for esophageal cancer. While rehabilitation interventions aligned with Enhanced Recovery After Surgery (ERAS) principles show promise, the term “rehabilitation” is often applied to highly variable approaches—from isolated breathing exercises to comprehensive multimodal programs—raising concerns about clinical interpretability and generalizability. This systematic review evaluates the effectiveness of structured perioperative rehabilitation, with emphasis on intervention comprehensiveness, timing, and implications for real-world implementation.

**Methods:**

We systematically searched PubMed, CINAHL, Cochrane Library, Web of Science, CNKI, Wanfang, and CBM from inception to October 31, 2024. Of 37 included studies, only 12 delivered interventions integrating ≥2 core components (e.g., exercise plus nutrition or education). Due to high clinical and statistical heterogeneity (*I^2^* > 90% for most outcomes), we prioritized narrative synthesis and conducted meta-analyses only within homogeneous subgroups—particularly those delivering continuous care across both preoperative and postoperative periods. Outcomes included functional capacity (6-min walk distance), cardiopulmonary function, pneumonia incidence, length of hospital stay (LOS), and health-related quality of life (HRQoL).

**Results:**

Comprehensive perioperative rehabilitation (integrating prehabilitation and postoperative rehabilitation) was uniquely associated with a significant reduction in postoperative pneumonia [RR = 0.34, 95% CI (0.19, 0.61); *p* < 0.0001]. In contrast, the pooled effect across all rehabilitation interventions showed a modest but statistically significant benefit [RR = 0.70, 95% CI (0.52, 0.96); *p* = 0.02], suggesting that while even simplified protocols may confer some protection, maximal risk reduction requires continuous, multimodal support spanning the entire surgical trajectory. In contrast, prehabilitation alone achieved the greatest reduction in hospital length of stay [MD = −2.94 days, 95% CI (−5.50, −0.37)] and significantly improved FEV₁ (SMD = 0.60). Multimodal programs consistently enhanced health-related quality of life [SMD = 0.76, 95% CI (0.60, 0.92)] and functional capacity (6-min walk distance, SMD = 0.90). Single-component interventions (e.g., respiratory training alone) showed inconsistent or negligible effects. The current evidence base is predominantly derived from studies conducted in China, reflecting strong regional research momentum while highlighting the need to validate these findings in other healthcare contexts.

**Conclusion:**

Multimodal, multi-phase rehabilitation may meaningfully improve short-term recovery after esophagectomy, but its benefits are outcome-specific and contingent on intervention design: pneumonia prevention requires integrated perioperative care, while reductions in resource use and improvements in baseline physiology are primarily driven by prehabilitation. The current literature suffers from conceptual dilution of “rehabilitation,” methodological variability, and limited long-term data. To enhance public health impact, future efforts should focus on scalable, standardized delivery models—such as community-integrated or telehealth-supported pathways—that extend support beyond hospital discharge, particularly in resource-constrained settings.

**Systematic review registration:**

PROSPERO registration number: CRD42024617815.

## 1 Introduction

Esophageal cancer (EC) is a malignant tumor arising from the epithelium of the esophagus and ranks as the eighth most common cancer and the sixth leading cause of cancer-related mortality worldwide (1). According to the World Health Organization, over 600,000 new cases and more than 540,000 deaths were attributed to EC globally in 2020, underscoring its substantial public health burden and the urgent need for effective interventions (2).

Surgery remains the cornerstone of curative treatment for EC. However, due to the complexity of the procedure—including extensive thoracoabdominal dissection, digestive tract reconstruction, chest wall musculature damage, and postoperative pain—patients are at high risk for postoperative complications. Pulmonary complications are particularly prevalent, with reported incidence rates ranging from 30 to 70%, including pneumonia, respiratory failure, pulmonary atelectasis, and pleural effusion (3). These complications not only prolong hospital stays and increase healthcare costs but also negatively impact long-term survival and quality of life (4).

To address these challenges, the Enhanced Recovery After Surgery (ERAS) program has emerged as a promising multimodal approach. First introduced by Danish surgeon Henrik Kehlet in 2002, ERAS is a patient-centered, evidence-based, multidisciplinary care pathway that integrates optimized perioperative practices in anesthesia, surgery, nursing, nutrition, and rehabilitation to reduce surgical stress responses, accelerate recovery, and minimize complications (5).

Since the formation of the ERAS Society in 2010, standardized guidelines have been developed across various surgical specialties, including colorectal, bariatric, gastric, hepatic, and gynecologic oncology procedures. These guidelines have consistently demonstrated benefits in reducing hospitalization duration and complication rates without compromising safety (6).

In recent years, ERAS-based rehabilitation strategies have increasingly been applied to patients undergoing esophagectomy. A meta-analysis by Pisarska et al., which included 2,042 patients, found that implementation of ERAS protocols significantly reduced both the length of hospital stay and the incidence of postoperative pulmonary complications (7). In 2018, the ERAS Society published specific guidelines for perioperative care in esophagectomy, aiming to standardize clinical practice and improve outcomes (8).

Within this context, it is essential to distinguish between related but distinct rehabilitation paradigms.

- Prehabilitation refers to structured, multimodal interventions delivered *before surgery* with the goal of enhancing physiological reserve, improving functional capacity, and mitigating postoperative morbidity. Core components often include aerobic and resistance exercise, inspiratory muscle training, nutritional optimization, and psychological support.
- Postoperative rehabilitation, in contrast, encompasses therapeutic strategies initiated *after surgery* to restore mobility, respiratory function, and activities of daily living, typically involving early mobilization, chest physiotherapy, and progressive exercise.
- Comprehensive perioperative (or multimodal) rehabilitation integrates both prehabilitation and postoperative rehabilitation into a continuous, coordinated care pathway spanning the entire surgical journey—from diagnosis through recovery.

Despite growing interest in ERAS-based rehabilitation, significant heterogeneity persists in the design, delivery, and evaluation of these programs across studies. This includes variations in intervention components (e.g., type, intensity, and timing of exercises), outcome measures (e.g., functional capacity, pulmonary function, quality of life), and study populations (9–11). Critically, many studies conflate “prehabilitation” with general “rehabilitation,” or fail to specify whether interventions were delivered preoperatively, postoperatively, or across both phases. This variability in intervention content, timing (preoperative-only vs. multi-phase), duration, and outcome assessment complicates interpretation of pooled effects and limits clinical applicability.

Given these challenges, this systematic review and meta-analysis aims to critically evaluate the current evidence on rehabilitation interventions for patients undergoing esophageal cancer surgery. We explicitly differentiate interventions by phase (preoperative, postoperative, or combined) and modality (single-component vs. multimodal). By clarifying which components and sequences are most consistently associated with improved functional capacity, reduced complications, and better quality of life, we aim to provide clinicians and perioperative teams with practical, evidence-informed guidance for optimizing post-esophagectomy recovery.

## 2 Materials and methods

This study was conducted in accordance with the Preferred Reporting Items for Systematic Reviews and Meta-Analyses (PRISMA 2020) statement (12) (see Supplementary Table S1 for checklist). The study protocol was prospectively registered in the International Prospective Register of Systematic Reviews (PROSPERO, CRD42024617815; https://www.crd.york.ac.uk/PROSPERO/view/CRD42024617815).

### 2.1 Study selection

A comprehensive systematic literature search was conducted across the following electronic databases: PubMed, Web of Science, Embase, and CINAHL for English-language studies; and CNKI, Wanfang Data, and SinoMed for Chinese-language studies. The search covered all records from database inception up to October 31, 2024. Boolean operators were used to combine keywords and free-text terms related to “esophagectomy,” “physical therapy,” “exercise therapy,” “muscle training,” “mobilization,” and “respiratory techniques.” Relevant synonyms and closely related terms were also included. In addition, reference lists of included articles were manually screened to identify potentially missed studies. The full search strategy is detailed in Supplementary Table S2.

### 2.2 Inclusion and exclusion criteria

Studies were considered eligible for inclusion in the meta-analysis if they met the following PICO-based criteria: i) Type of study: Randomized controlled trials (RCTs), quasi-experimental studies, cohort studies, or other primary interventional studies; ii) Population: Adult patients (≥18 years) who had undergone esophageal cancer surgery; iii) Intervention: Any form of rehabilitation strategy, including respiratory training, aerobic exercise, resistance training, or mobilization; iv) Outcomes: At least one outcome related to physical function or postoperative complications (e.g., pulmonary complications, hospital length of stay, quality of life, or exercise capacity); v) Language: Published in either Chinese or English.

Exclusion criteria were as follows: i) Duplicate publications or overlapping data; ii) Abstract-only publications or inaccessible full texts; iii) non-empirical literature (e.g., reviews, guidelines, conference abstracts, protocols, or case reports).

### 2.3 Data extraction

Two independent reviewers, trained in evidence-based methodology, conducted literature screening and data extraction. Discrepancies were resolved through discussion, with a third reviewer consulted when consensus could not be reached. A standardized data extraction form was developed to collect information on study characteristics (author, year, journal, title, study type), population (sample size), intervention details (timing, modality), and outcomes (name, measurement time point, assessment tool).

### 2.4 Statistical analysis

Meta-analyses were conducted only for outcomes reported by at least two independent studies with a concurrent control or comparison group. The primary evidence base consisted of randomized controlled trials (RCTs). When fewer than two RCTs were available for a specific outcome, non-randomized comparative studies (e.g., prospective cohorts with controls) were considered to inform a narrative synthesis; however, they were not pooled with RCTs in the main meta-analysis unless explicitly stated in sensitivity analyses. Single-arm studies and non-comparative designs were excluded from all quantitative syntheses and summarized descriptively.

For continuous outcomes measured on the same scale across studies (e.g., 6-min walk distance in meters, hospital length of stay in days, FEV₁ in liters), the weighted mean difference (WMD) with 95% confidence intervals (CIs) was calculated. When outcomes were assessed using different instruments or incomparable units (e.g., health-related quality of life measured by SF-36, EORTC QLQ-C30, or EQ-5D), the standardized mean difference (SMD) was used. All effect estimates were interpreted according to conventional benchmarks (e.g., Cohen’s thresholds for SMD: 0.2 = small, 0.5 = moderate, 0.8 = large).

For dichotomous outcomes (e.g., postoperative pneumonia, mortality), risk ratios (RR) with 95% CIs were used as the primary effect measure, particularly given the generally low event rates (<10%) in most included studies, under which RR provides a clinically intuitive estimate of relative risk.

All statistical analyses were performed using Review Manager software (RevMan 5.4, Cochrane Collaboration). Missing or incomplete data were addressed by contacting original study authors when feasible; if unavailable, no imputation was performed, and available-case analysis was applied.

### 2.5 Heterogeneity assessment

Statistical heterogeneity across studies was evaluated using Cochran’s Q test (with significance set at *p* < 0.10) and quantified using the *I^2^* statistic. An *I^2^* value greater than 50% was interpreted as indicating substantial heterogeneity. In such cases, a random-effects model (DerSimonian and Laird method) was used to account for between-study variability. When heterogeneity was low (*I^2^* ≤ 50% and *p* ≥ 0.10 for the *Q* test), a fixed-effect model was applied.

Publication bias was assessed visually via funnel plots and statistically using Egger’s regression test, where at least 10 studies were available for a given outcome. Pre-specified subgroup analyses were conducted according to intervention timing [preoperative only, postoperative only, or perioperative (pre plus postoperative)] and study design (RCT vs. non-randomized studies).

### 2.6 Risk of Bias assessment

The methodological quality and risk of bias in included RCTs were assessed using the Cochrane risk-of-bias tool for randomized trials (RoB 1.0) (13). Six domains were evaluated: sequence generation, allocation concealment, blinding of participants and personnel, blinding of outcome assessors, incomplete outcome data, and selective reporting. Each domain was judged as low, high, or unclear risk of bias. The overall risk of bias was determined based on the domain-level judgments.

## 3 Results

### 3.1 Description of the studies

**Figure 1:**
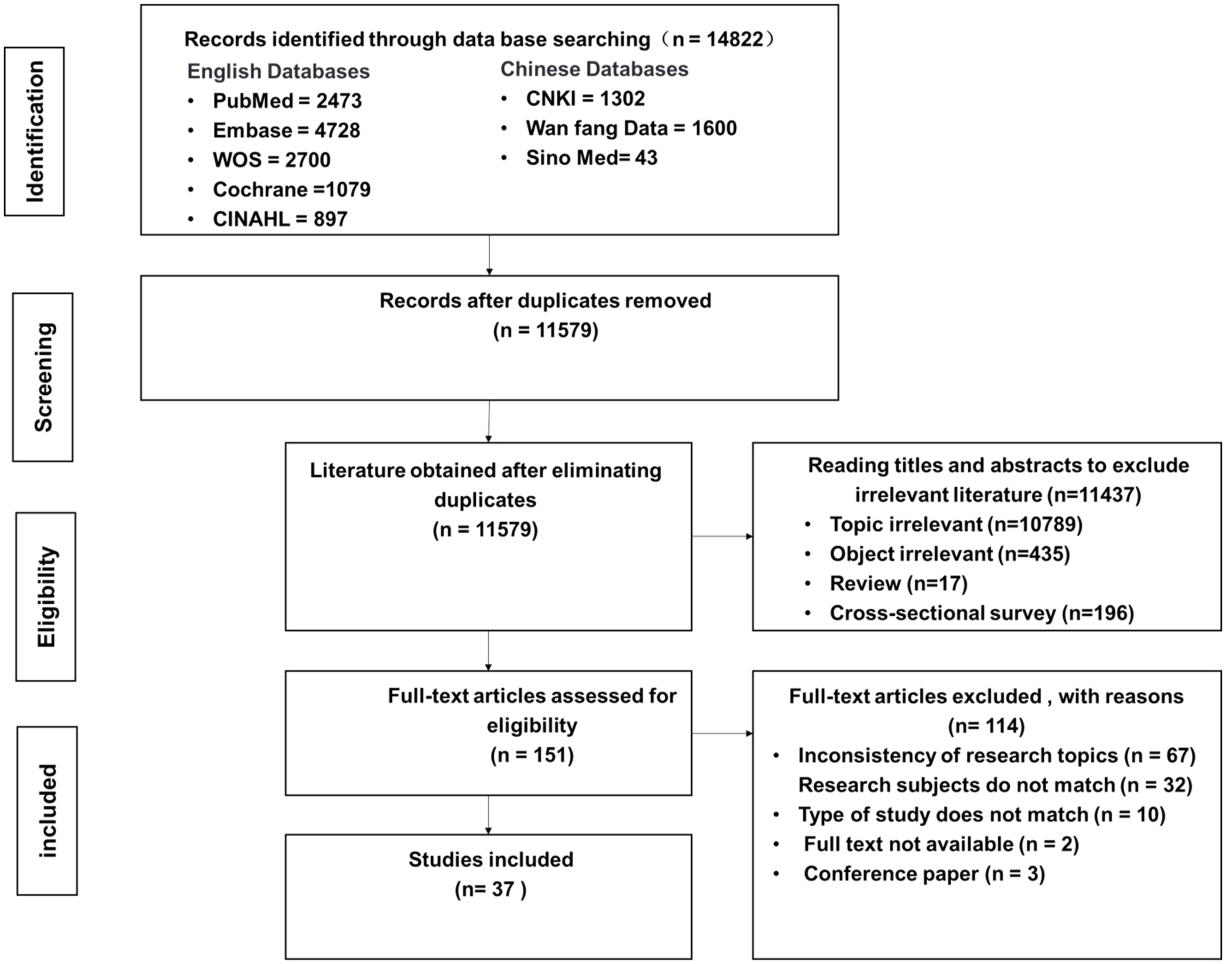
PRISMA flow diagram.

In this study, an initial search yielded 14,822 documents. After applying de-duplication using Note Express and conducting title and full-text screening, a total of 37 documents were ultimately included in the analysis. The detailed literature screening process is illustrated in Figure 1.

A total of 37 studies were included in this review, comprising 26 randomized controlled trials (RCTs), 3 pilot RCTs, 5 prospective cohort studies, and 3 single-arm studies. The studies spanned multiple countries and regions, including China, Ireland, the United Kingdom, Japan, the Netherlands, France, South Korea, and Canada. The overall sample size across all included studies was 2,216 participants. Detailed baseline characteristics are presented in Table 1.

**Table 1:** Basic characteristics of the included literature (*n* = 37).

Authors (year)	Country	Topic of an article	Type	No. of patients
Loughney L et al. (2024) (25)	Ireland	A pre and post-operative exercise program	RCT	36/35
Xuelan P et al. (2024) (19)	China	Respiratory muscle resistance training	RCT	37/37
Chen Lihua et al.(2024) (14)	China	Rehabilitation training combined with assisted vehicle	RCT	30/30
Qian Yan et al. (2023) (20)	China	Application of prehabilitation strategy	RCT	40/40
Ma Ling et al. (2023) (33)	China	Rapid rehabilitation mode combined with vibration sputum removal	RCT	53/53
LI Shiyu et al. (2022) (34)	China	Vibrating sputum expectorator combined with comprehensive respiratory function training	RCT	46/46
Allen SK et al. (2022) (21)	UK	Multimodal prehabilitation	RCT	27/27
Shen et al. (2022) (35)	China	Enhanced recovery	RCT	60/58
Do JH et al. (2022) (36)	South Korea	Multimodal inpatient rehabilitation vs. conventional pulmonary rehabilitation	RCT	29/30
van V et al. (2017) (37)	Netherlands	Supervised exercise	RCT	61/59
Ai-Ying S et al. (2021) (15)	China	Quality-control circle activities in respiratory function exercise	RCT	48/48
Wang X. (2021) (18)	China	Triple prehabilitation strategy	RCT	44/44
Hui-Yu J et al. (2019) (16)	China	Target exercise combined with multifunctional chest belt for rapid rehabilitation care	RCT	68/68
Jin ling G et al. (2019) (38)	China	Quantitative goal exercise on rapid rehabilitation	RCT	122/122
Meiling J et al. (2019) (39)	China	A three-position integrated respiratory management model for health care providers and patients	RCT	33/32
Guinan et al. (2019) (28)	Ireland	Preoperative inspiratory muscle training	RCT	28/32
Minnella et al. (2018) (22)	Canada	Exercise and nutrition prehabilitation	RCT	26/25
Lu Li et al. (2017) (40)	China	Respiratory training	RCT	40/40
Huang XY et al. (2017) (26)	China	Inspiratory muscle training	RCT	45/45
O’Neill et al. (2017) (41)	Ireland	Multidisciplinary rehabilitative program	RCT	21/22
Valkenet K et al. (2017) (42)	Netherlands	Inspiratory muscle training	RCT	120/121
Yamana I et al. (2015) (43)	Japan	A preoperative respiratory rehabilitation program	RCT	30/30
Chen WJ et al. (2014) (31)	China	Active breathing training	RCT	20/20
Chen W et al. (2013) (29)	China	Breathing training	RCT	30/30/30
Aichun P et al. (2009) (44)	China	Respiratory function exercise	RCT	27/26
Wanxia W et al. (2006) (45)	China	Rehabilitation training	RCT	36/35
Haiqing Z et al. (2022) (30)	China	Nurse-led triple prehabilitation management model	Quasi-RCT	44/48
Deng YP et al. (2011) (17)	China	Respiratory function training	Quasi-RCT	100/100
Li W et al. (2017) (27)	China	Modified enhanced recovery	Quasi-RCT	55/55
Akiyama et al. (2021) (46)	Japan	Efficacy of enhanced prehabilitation	Cohort	31/21
Halliday LJ et al. (2023) (32)	UK	Multimodal therapy	Cohort	51/28
Ikeda et al. (2022) (47)	Japan	Early exercise	Cohort	71/39
Halliday et al. (2020) (48)	UK	Adherence to pre-operative exercise	Cohort	67/−−
Lococo et al. (2012) (23)	Italy	Intensive long-term pulmonary rehabilitation program	Cohort	50/8
Chmelo J et al. (2022) (49)	UK	A multi-component home-based exercise programme	Single-arm	39/−−
Yang K et al. (2021) (50)	Korea	An interactive health coaching mobile app	Single-arm	36/−−
Kenneth et al. (2021) (24)	Canada	Multimodal prehabilitation	Single-arm	22/21

RCT, Randomized Controlled Trial; quasi-RCT, Quasi-randomized Controlled Trial; cohort, Cohort Study; single-arm, Single-Arm Study.

### 3.2 Characteristics of the rehabilitation intervention

**Figure 2:**
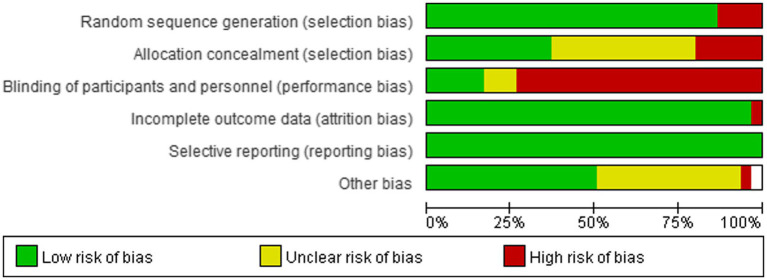
Risk of bias assessment.

Among the 37 included studies, a diverse range of exercise-based prehabilitation and postoperative rehabilitation interventions were reported. These interventions primarily encompassed respiratory exercises, aerobic and endurance training, and resistance training. Collectively, they aimed to enhance patients’ physical capacity, optimize perioperative recovery, and reduce the risk of postoperative complications. The key characteristics of each type of intervention are summarized below Figure 2.

#### 3.2.1 Respiratory exercises

Respiratory exercises were described in 22 studies and included techniques such as pursed-lip breathing, diaphragmatic breathing, deep breathing, effective coughing, balloon blowing, and the use of respiratory trainers. These interventions typically commenced 1–2 weeks before surgery, with session duration gradually increasing from 5–10 min per session to 15–20 min, performed 2–3 times daily, depending on individual patient tolerance.

Four studies (14) (15) (16) (17) combined respiratory exercises with active or passive limb movements to further promote circulation and prevent postoperative complications such as venous thromboembolism. One study (18) provided a comprehensive description of its prehabilitation protocol, which also incorporated individualised nutritional support plans to improve patients’ nutritional status and optimise surgical outcomes.

To enhance adherence to the respiratory training program, one research group (19) developed instructional videos, which significantly improved patient compliance. Improvements were observed in several pulmonary function parameters, including maximum inspiratory pressure (MIP), maximum expiratory pressure (MEP), peak expiratory flow (PEF), forced vital capacity (FVC), and forced expiratory volume in 1 second (FEV1).

#### 3.2.2 Aerobic exercise and endurance training

A total of 15 studies focused on aerobic and endurance training, involving activities such as breathing gymnastics, brisk walking, jogging, and cycling. Five studies (20–24) initiated their aerobic exercise programs 1–2 weeks preoperatively with the goal of enhancing cardiopulmonary function.

Initial sessions lasted 20–30 min, gradually increasing to 30–60 min per session, and were conducted 3–5 times per week. One study (23) implemented an example of a long-term aerobic intervention, offering a personalised, home-based preoperative exercise program lasting up to 16 weeks, requiring participants to achieve at least 600 MET-minutes per week of moderate-intensity activity. This program was supported by self-reporting tools and telephone follow-ups to promote adherence.

Eight studies incorporated postoperative aerobic exercise regimens into their intervention protocols. For instance, one study (25) offered either a centre-based exercise program (CBEP) led by ExWell Medical across seven centres or a home-based exercise program (HBEP), starting from cancer diagnosis through 6 weeks post-surgery. In another study, one group (26) extended the exercise intervention for up to 1 year after surgery.

#### 3.2.3 Resistance training

Four studies (16, 19, 20, 27) integrated resistance training into their prehabilitation programs alongside respiratory and aerobic exercises to enhance overall effectiveness. The resistance training components were designed to improve muscular strength and endurance through two main approaches:

- *Basic resistance exercises*: These involve body-weight exercises such as stair climbing, squats, and straight leg raises in bed, which are effective for strengthening lower limb and core muscles.
- *Resistance training with assistive equipment*: Tools such as resistance bands, dumbbells, grip trainers, and sandbags were used to add resistance and variety to upper-body muscle training, promoting more comprehensive strength development and stability.

By integrating multiple types of training modalities, these studies addressed not only cardiorespiratory function and respiratory technique improvement but also emphasised muscle strength and overall physical conditioning. Such a multimodal approach aims to comprehensively enhance patients’ preoperative functional status and accelerate the rehabilitation process.

The detailed characteristics of the rehabilitation interventions are presented in Table 2.

**Table 2:** Characteristics of perioperative rehabilitation interventions by timing.

Type of exercise	Specific measures	Timing	Study (year)
Respiratory Exercises	Pursed-lip breathing, abdominal breathing, deep breathing, effective coughing, blowing balloons, and respiratory trainer exercises	Preoperative only	Qian Yan et al. (2023) (20) Ma Ling et al. (2023) (33) Aichun P et al. (2009) (44) Haiqing Z et al. (2022) (30) Guinan et al. (2019) (28)
		Postoperative only	Xuelan P et al. (2024) (19) Ai-ying S et al. (2021) (15) Hui-yu J et al. (2019) (16) Gu Jinling et al. (2019) (38)
			Lu Li et al. (2017) (40) Huang XY et al. (2017) (26) Chen WJ et al. (2014) (31)
		Perioperative (Pre + Post)	Li Shiyu et al. (2022) (34) Wang Wanxia et al. (2006) (45) Shen et al. (2022) (35) Meiling J et al. (2019) (39) Li Wei et al. (2017) (27)
Aerobic exercise and endurance training	breathing gymnastics, brisk walking, jogging, and cycling	Preoperative only	Qian Yan et al. (2023) (20) Allen SK et al. (2022) (21) Minnella et al. (2018) (22) Halliday et al. (2023) (32) Halliday et al. (2021) (48) Yang et al. (2021) (50) Kenneth et al. (2021) (24)
		Postoperative only	Do JH et al. (2022) (36) van V et al. (2017) (37) O’Neill et al. (2018) (41) Ikeda et al. (2022) (47) Lococo et al. (2012) (23)
		Perioperative (Pre + Post)	Loughney L et al. (2024) (25) Shen et al. (2022) (18) Meiling J et al. (2019) (39) Akiyama et al. (2021) (46)
Resistance training	Upper limb muscle training using dumbbells, grip trainers, or resistance bands; lower limb muscle training through stair climbing, squats, bed leg lifts	Preoperative only	Qian Yan et al. (2023) (20) Wang X. (2021) (18) Haiqing Z et al. (2022) (30) Halliday et al. (2023) (32) Halliday et al. (2021) (48) Kenneth et al. (2021) (24)
		Postoperative only	Hui-Yu J et al. (2019) (16) Jin ling G et al. (2019) (38) O’Neill et al. (2018) (41) Valkenet K et al. (2017) (42) Ikeda et al. (2022) (47) Lococo et al. (2012) (23) Chmelo et al. (2022) (49)
		Perioperative (Pre + Post)	CHEN Lihua et al. (2024) (14) Wang Wanxia et al. (2006) (30) Li W et al. (2017) (27) Akiyama et al. (2021) (46)

### 3.3 Risk of bias and publication bias assessment

**Figure 3:**
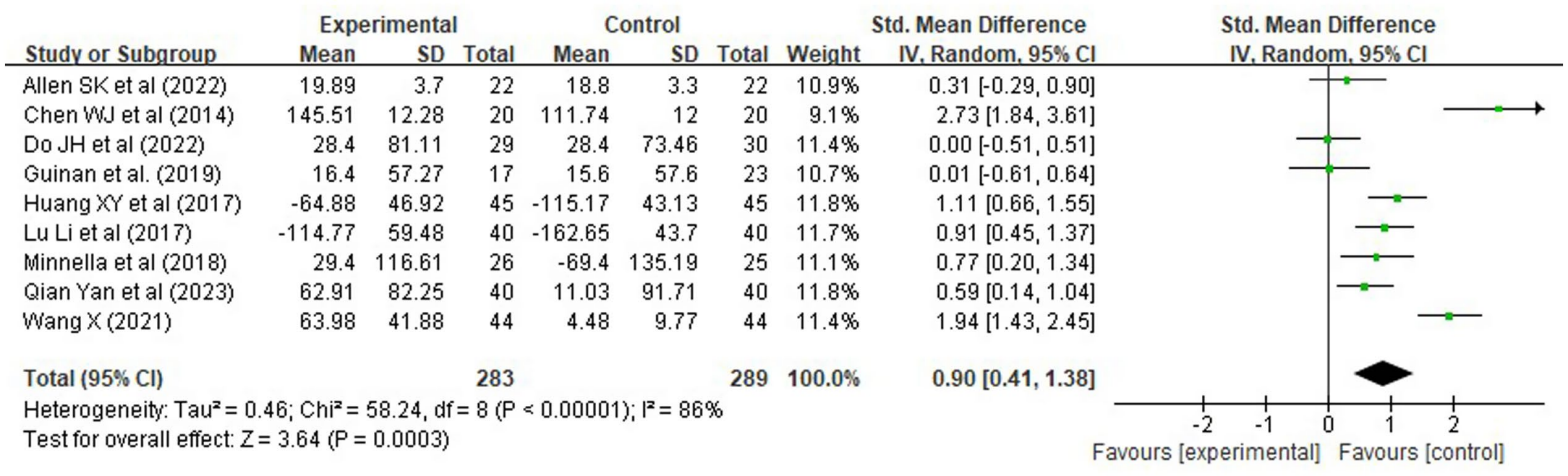
Forest plot of rehabilitation effect on 6MWT.

The risk of bias among the included randomized controlled trials (RCTs) is summarized in Figure 3. Overall, the studies exhibited a low risk of bias across key domains, including random sequence generation, allocation concealment, completeness of outcome data, and selective reporting. However, a high or unclear risk of bias was observed in relation to blinding of participants and personnel, as well as other potential sources of bias.

These findings highlight the need for future research to prioritize methodological rigor—particularly through improved implementation of blinding procedures and careful control of confounding variables—to further enhance the quality and reliability of evidence in this field.

Publication bias was assessed using funnel plots. Symmetry in the funnel plots suggested minimal publication bias for the primary outcomes; however, due to the limited number of studies included in each analysis, these results should be interpreted with caution.

### 3.4 Meta-analysis results by intervention timing

#### 3.4.1 Functional exercise capacity (6-minute walk test)

A total of 10 randomized controlled trials (RCTs), involving 572 patients, reported outcomes on functional exercise capacity using the 6-min walk test (6MWT). Given the variability in assessment timing—ranging from preoperative baseline to postoperative recovery periods—the standardized mean difference (SMD) was employed as the effect size metric to enable meaningful pooling. Pooled analysis revealed that rehabilitation interventions significantly improved 6MWT distance, with an SMD of 0.90 [95% CI (0.41, 1.38), Z = 3.64, *p* = 0.0003], corresponding to a large effect size according to Cohen’s criteria (*d* > 0.8). This indicates a substantial benefit of structured rehabilitation programs on functional mobility (Figure 3).

**Figure 4:**
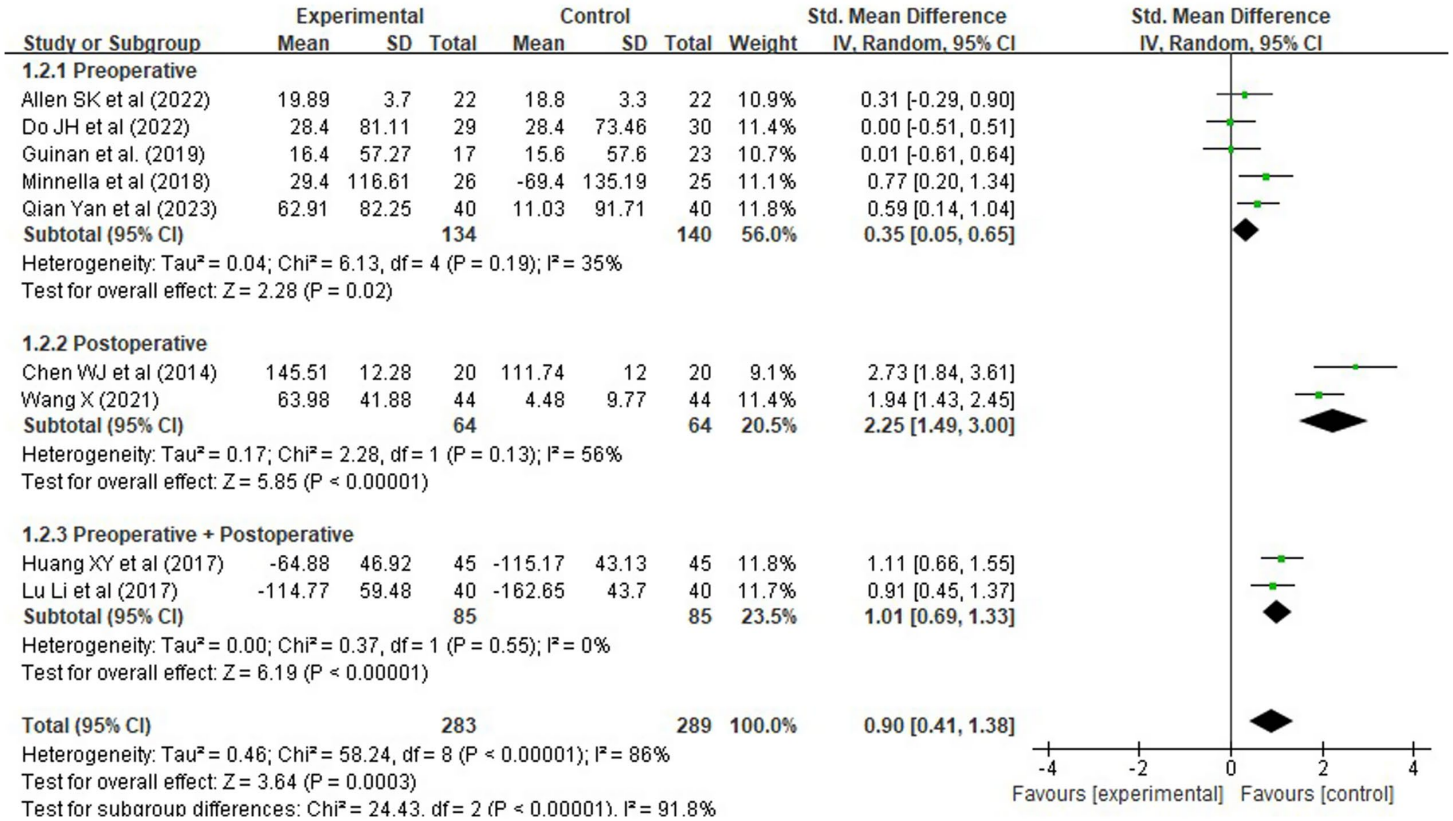
Forest plots of rehabilitation effect on 6MWT by intervention timing.

However, substantial heterogeneity was observed across studies (*χ^2^* = 58.24, df = 8, *p* < 0.00001; *I^2^* = 86%), likely attributable to differences in intervention design, intensity, and assessment timing. To explore the impact of intervention timing, a subgroup analysis was conducted based on whether the intervention was delivered preoperatively, postoperatively, or both (Figure 4). Results showed that:

- Comprehensive perioperative rehabilitation (pre plus postoperative) yielded the largest improvement in walking capacity [SMD = 1.01, 95% CI (0.69, 1.33), *p* < 0.00001], with no heterogeneity (*I^2^* = 0%), indicating consistent and robust benefits.
- Postoperative-only rehabilitation also demonstrated significant effects [SMD = 2.25, 95% CI (1.49, 3.00), *p < 0.00001*], though with high heterogeneity (*I^2^* = 56%).
- Preoperative-only interventions showed a statistically significant but modest improvement [SMD = 0.35, 95% CI (0.05, 0.65), *p* = 0.02], suggesting limited clinical benefit when applied in isolation.

To assess the robustness of these findings, a sensitivity analysis was conducted by excluding studies with small sample sizes (<20 participants), inadequate description of exercise protocols (e.g., missing details on frequency, duration, or intensity), or high risk of bias. Seven studies met these stricter criteria and were retained for re-analysis. The pooled effect remained statistically significant and slightly increased in magnitude [SMD = 0.94, 95% CI (0.43, 1.45), *p* = 0.0004], with heterogeneity remaining high but stable (*I^2^* = 82%). This indicates that the observed benefit of rehabilitation on functional exercise capacity is not driven by low-quality or poorly reported trials, but rather reflects a consistent effect among methodologically sound studies.

These findings underscore that integrated, multi-phase rehabilitation programs spanning both preoperative and postoperative periods are most effective in enhancing functional exercise capacity, offering greater clinical utility than isolated interventions.

#### 3.4.2 Cardiopulmonary function

**Figure 5:**
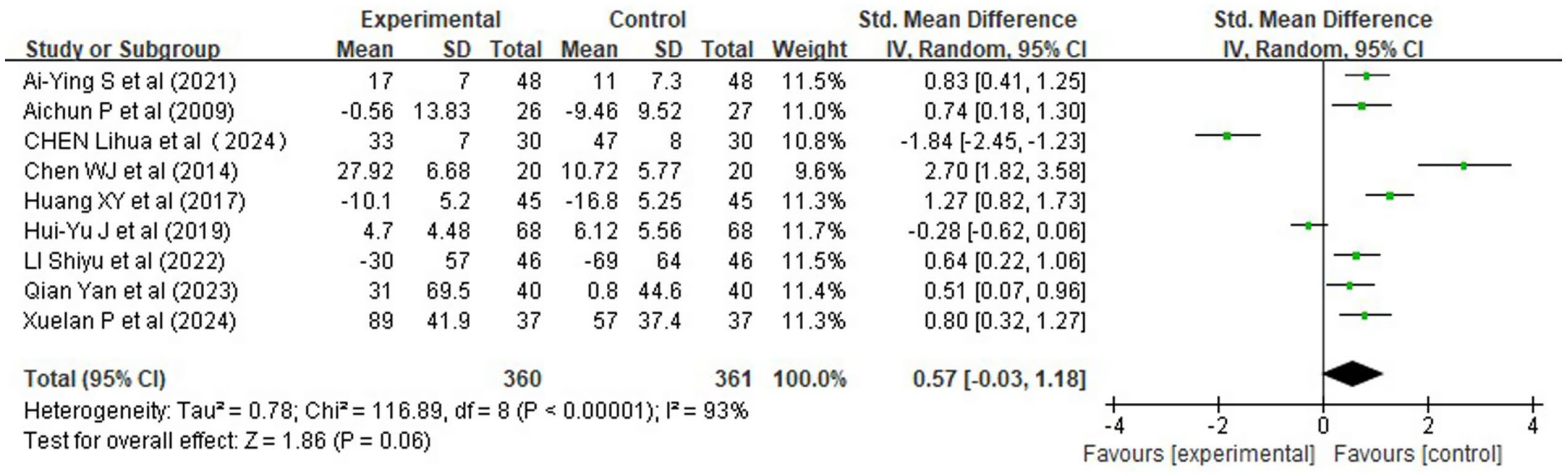
Forest plots of rehabilitation effect on cardiopulmonary function (SMD).

Nine randomized controlled trials (RCTs), involving 721 patients, reported changes in cardiopulmonary function, specifically forced expiratory volume in one second (FEV1) and the FEV1/FVC ratio (Figure 5). All studies reported change scores (postoperative minus preoperative values) expressed as percentages (%), with consistent units across trials. Given the variability in standard deviations (SDs) among studies, we used the standardized mean difference (SMD) as the effect size metric to enable meaningful pooling.

Pooled analysis revealed a non-significant trend toward improvement in respiratory function following rehabilitation interventions, with an overall SMD of 0.57 [95% CI (−0.03, 1.18), *Z* = 1.86, *p* = 0.06], indicating a small to moderate effect size according to Cohen’s criteria (*d* > 0.2). However, significant heterogeneity was present (*χ^2^* = 116.89, df = 8, *p* < 0.00001; *I^2^* = 93%), likely due to differences in intervention protocols, assessment timing, and baseline lung function.

**Figure 6:**
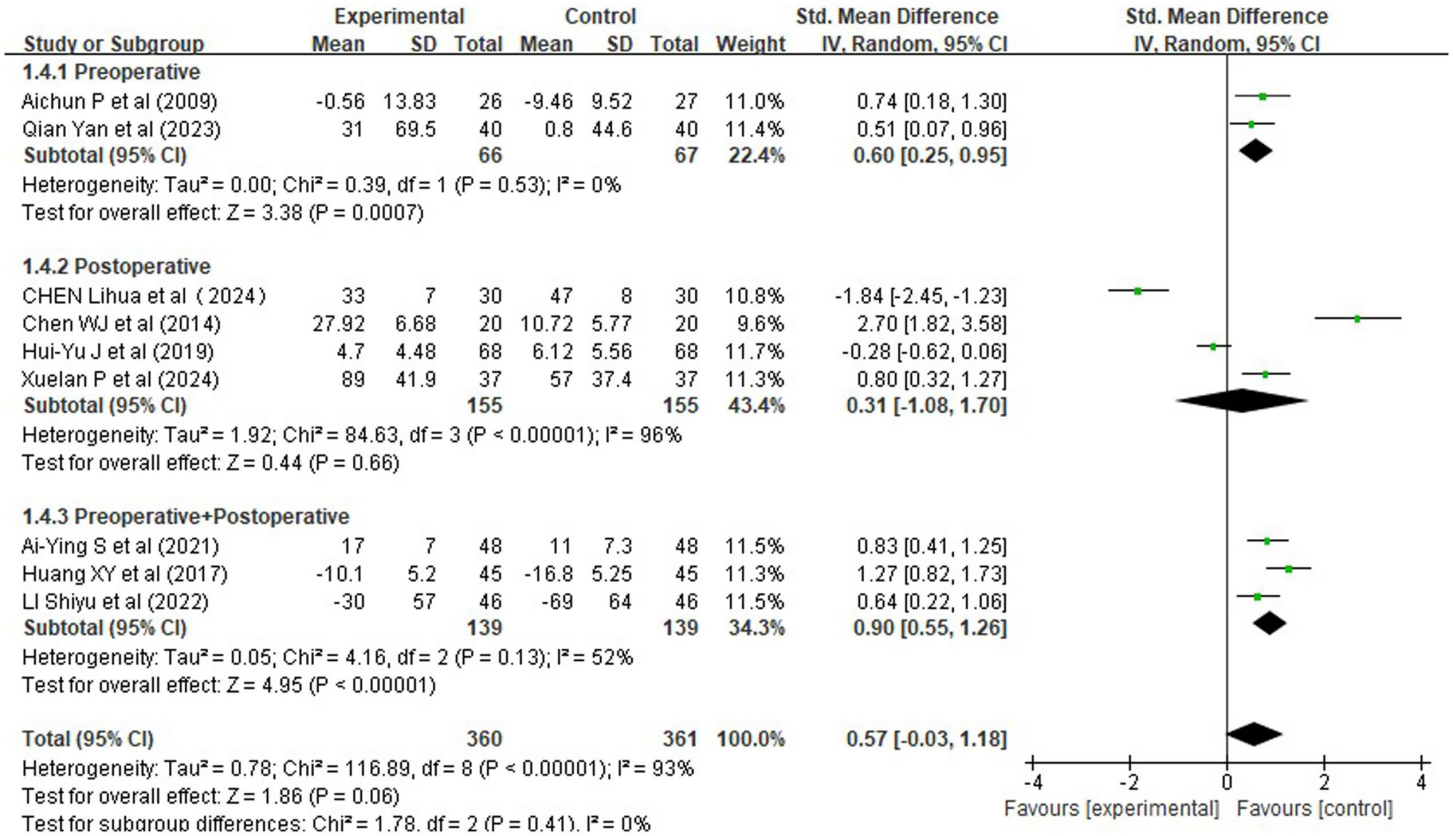
Forest plots of rehabilitation effect on cardiopulmonary function by intervention timing.

Subgroup analysis by intervention timing (Figure 6) showed that:

- Comprehensive perioperative rehabilitation (pre plus postoperative) yielded the most substantial benefit (SMD = 0.90, 95% CI [0.55, 1.26], *p* < 0.00001), with moderate heterogeneity (*I^2^* = 52%), suggesting robust improvement in pulmonary function.
- Preoperative-only interventions also demonstrated a significant effect [SMD = 0.60, 95% CI (0.25, 0.95), *p* = 0.0007], with no heterogeneity (*I^2^* = 0%), indicating consistent results across studies.
- Postoperative-only interventions did not reach statistical significance [SMD = 0.31, 95% CI (−1.08, 1.70), *p* = 0.66], although the direction favored the experimental group.

Collectively, these findings suggest that multi-phase rehabilitation strategies incorporating both preoperative and postoperative elements are more effective in preserving or enhancing cardiopulmonary function than single-phase approaches. The lack of benefit in postoperative-only groups may reflect limited duration or intensity of intervention. Future studies should aim to standardize protocols and define optimal timing and components to reduce heterogeneity and improve clinical translation.

#### 3.4.3 Pneumonia incidence: protective effect of combined pre and postoperative rehabilitation

**Figure 7:**
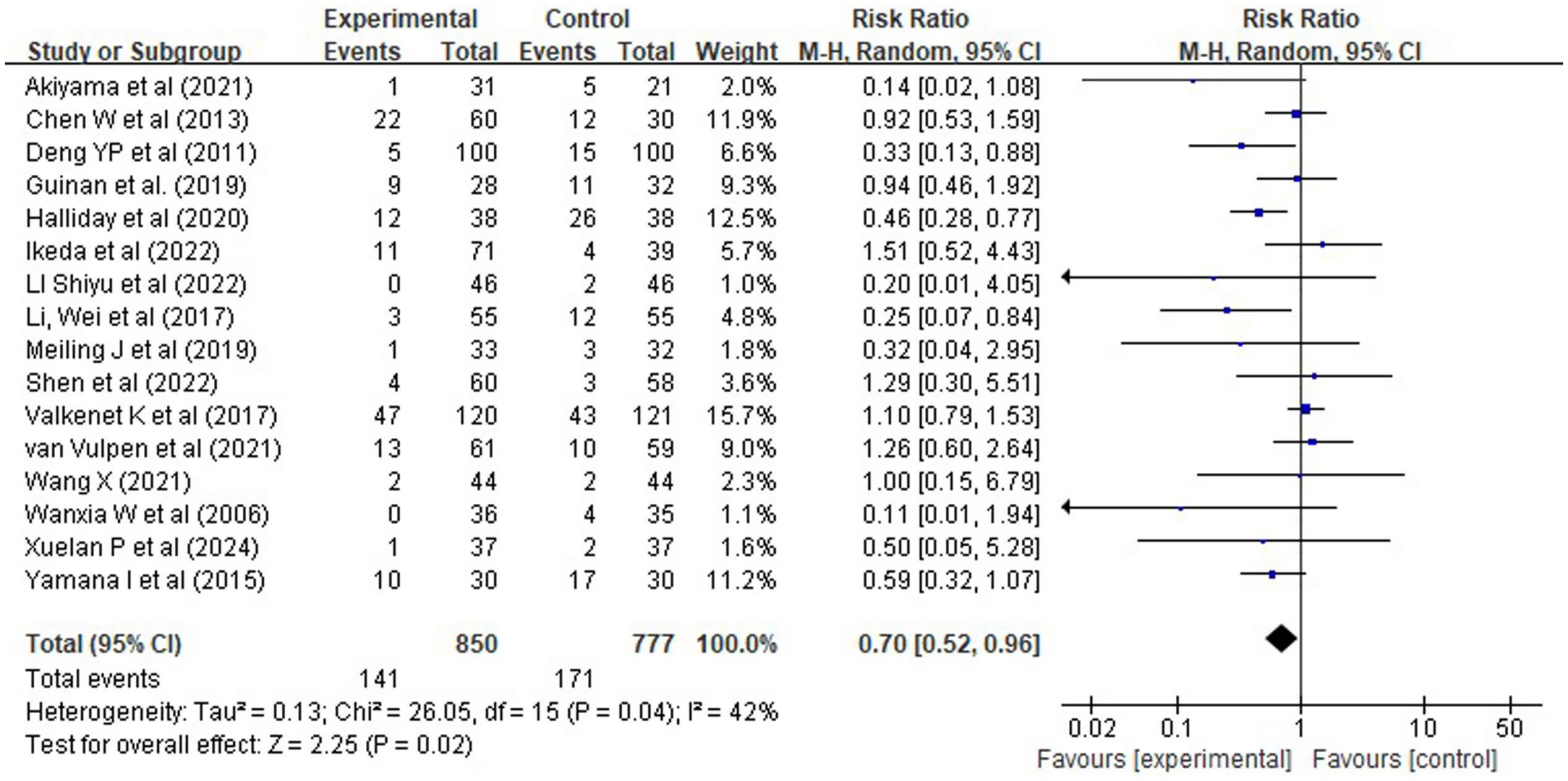
Forest plot of rehabilitation effect on pneumonia risk (RR).

Data from 14 randomized controlled trials reported pneumonia incidence in 850 patients in the experimental group and 777 in the control group, with 141 and 171 cases, respectively. Meta-analysis using a random-effects model indicated that rehabilitation interventions significantly reduced the risk of postoperative pneumonia [RR = 0.70, 95% CI (0.52, 0.96), *Z* = 2.25, *p* = 0.02] (Figure 7). Moderate heterogeneity was observed (*χ^2^* = 26.05, df = 15, *p* = 0.04; *I^2^* = 42%), likely attributable to differences in population characteristics, surgical procedures, or intervention components.

**Figure 8:**
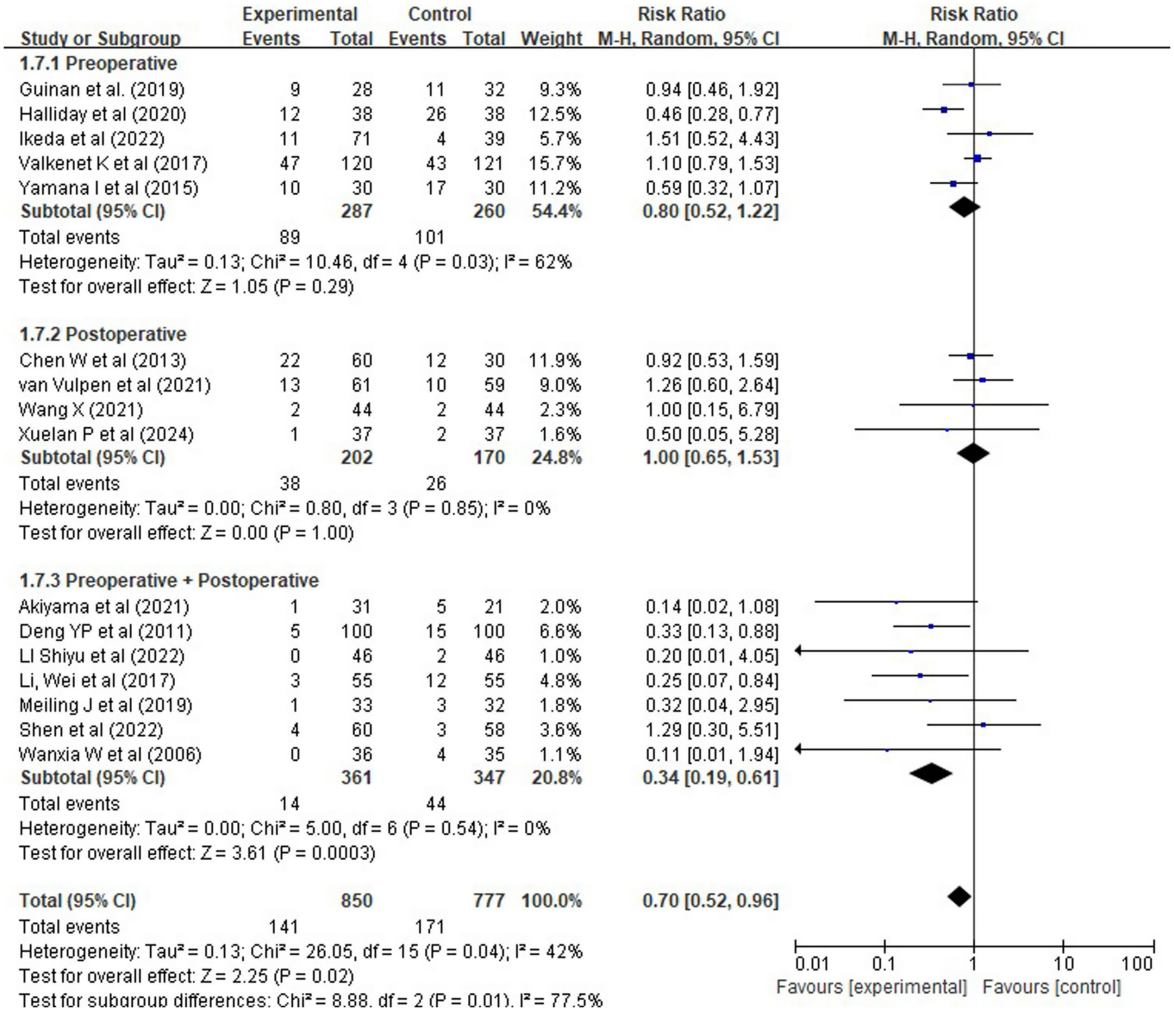
Forest plots of rehabilitation effect on pneumonia risk by intervention timing.

Subgroup analysis by intervention timing revealed that comprehensive perioperative rehabilitation (prehabilitation plus postoperative rehabilitation) had the strongest protective effect against pneumonia [RR = 0.34, 95% CI (0.19, 0.61), Z = 3.61, *p* < 0.0001], with no heterogeneity (*I^2^* = 0%). In contrast, neither prehabilitation alone [RR = 0.80, 95% CI (0.52, 1.22), *p* = 0.29] nor postoperative rehabilitation alone [RR = 1.00, 95% CI (0.65, 1.53), *p* = 1.00] demonstrated a statistically significant reduction in pneumonia risk. The absence of benefit with single-phase strategies suggests that continuity of care across the perioperative period may be essential for meaningful risk reduction Figure 8.

**Figure 9:**
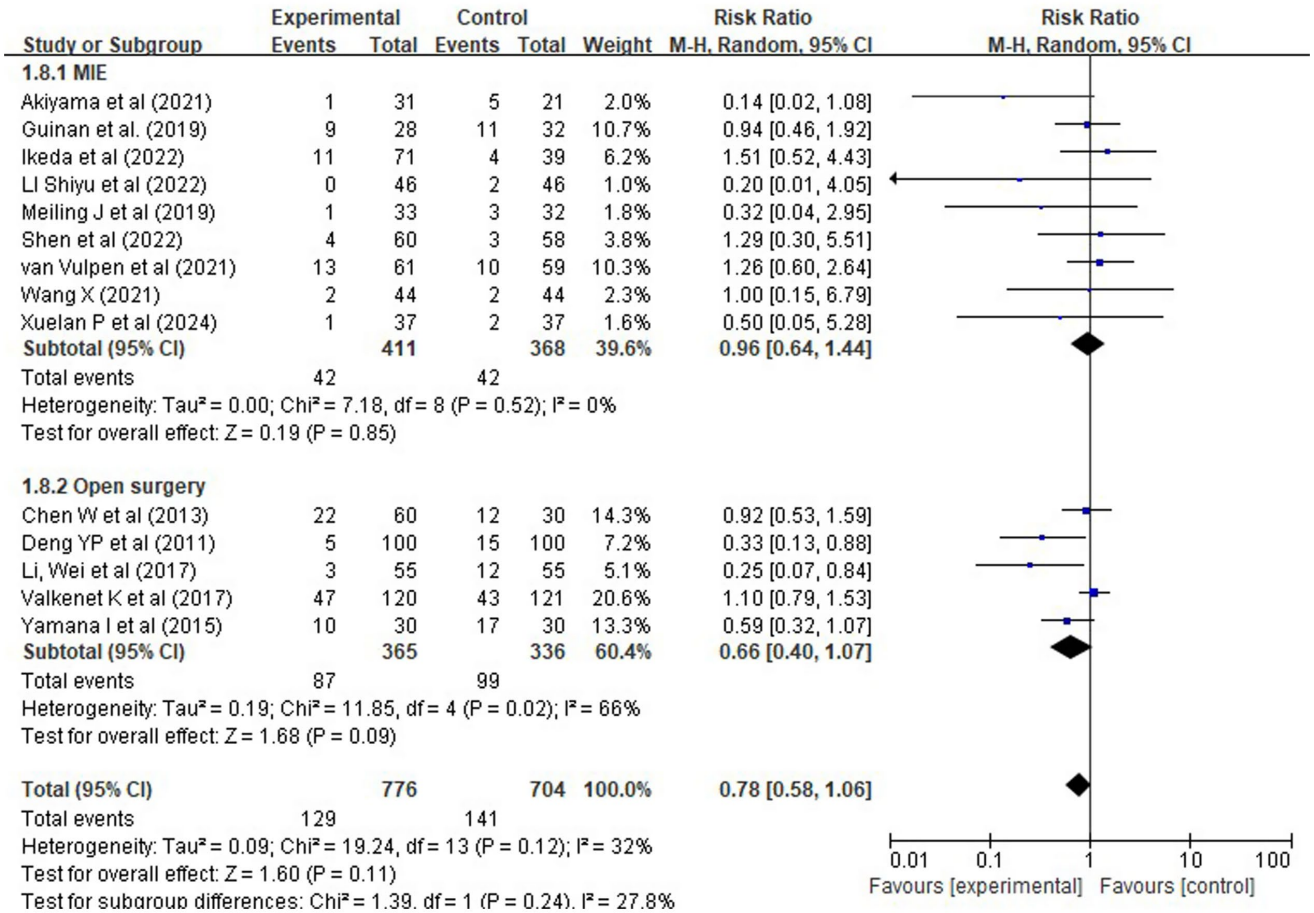
Forest plots of rehabilitation effect on pneumonia risk by surgical approach.

To explore whether surgical approach contributed to heterogeneity, we conducted a subgroup analysis among the 14 studies reporting procedure type: 9 used minimally invasive esophagectomy (MIE) and 5 used open surgery. The direction of effect was consistent across both subgroups (RR = 0.96 for MIE, RR = 0.66 for open surgery), with no significant interaction (*χ^2^* = 1.39, df = 1, *p* = 0.24; *I^2^* = 27.8%), suggesting that the benefit of comprehensive rehabilitation is not materially modified by surgical technique Figure 9.

Sensitivity analyses excluding four low-quality or ambiguously reported studies—defined as those with sample size <20, unclear intervention description, or high risk of bias—were performed. The pooled risk ratio remained significant [RR = 0.71, 95% CI (0.53, 0.95), Z = 2.35, *p* = 0.02], with similar heterogeneity (*I^2^* = 41%) and no change in the direction of effect. This consistency supports the robustness of our findings.

These findings suggest that comprehensive perioperative rehabilitation is associated with a substantially lower risk of postoperative pneumonia, with effects that are consistent and reproducible across studies. Given the moderate certainty of the evidence (per GRADE assessment), these results should not be interpreted as indicating a life-saving effect, but rather as supporting a clinically meaningful reduction in a common and morbid complication. Future high-quality RCTs are needed to confirm these results and establish optimal protocols.

#### 3.4.4 Length of hospital stay

**Figure 10:**
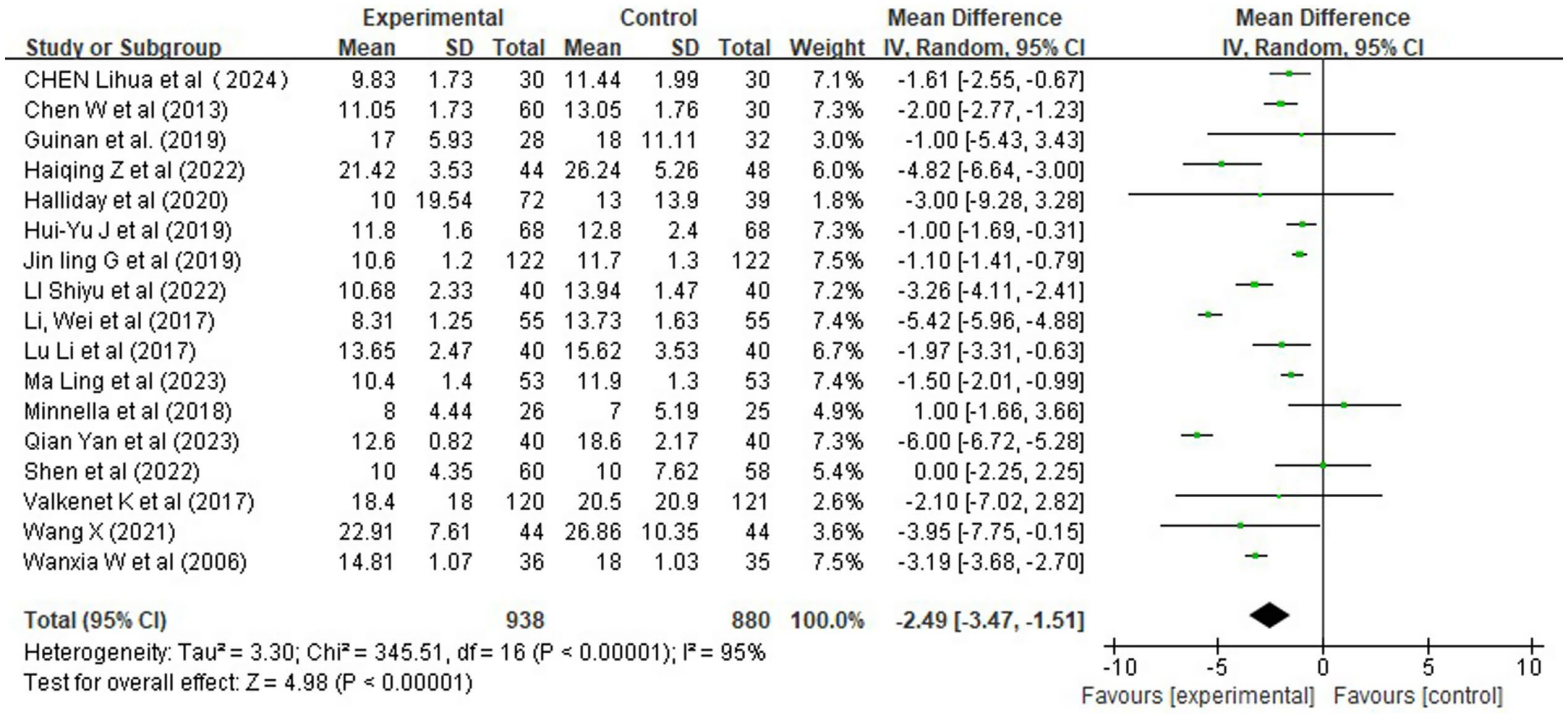
Forest plot of rehabilitation effect on length of hospital stay (MD).

A total of 18 studies evaluated the impact of prehabilitation and rehabilitation interventions on the length of hospital stay (Figure 10). Using a random-effects model to account for expected heterogeneity, meta-analysis revealed a statistically significant reduction in hospitalization duration in the intervention group compared with the control group: mean difference (MD) = −2.49, 95% CI [−3.47, −1.51], *Z* = 4.98, *p* < 0.00001.

However, substantial statistical heterogeneity was observed across studies (*χ^2^* = 345.51, df = 16, *p* < 0.00001; *I^2^* = 95%), likely attributable to variations in study populations, surgical procedures, intervention components, and definitions of hospital discharge criteria.

**Figure 11:**
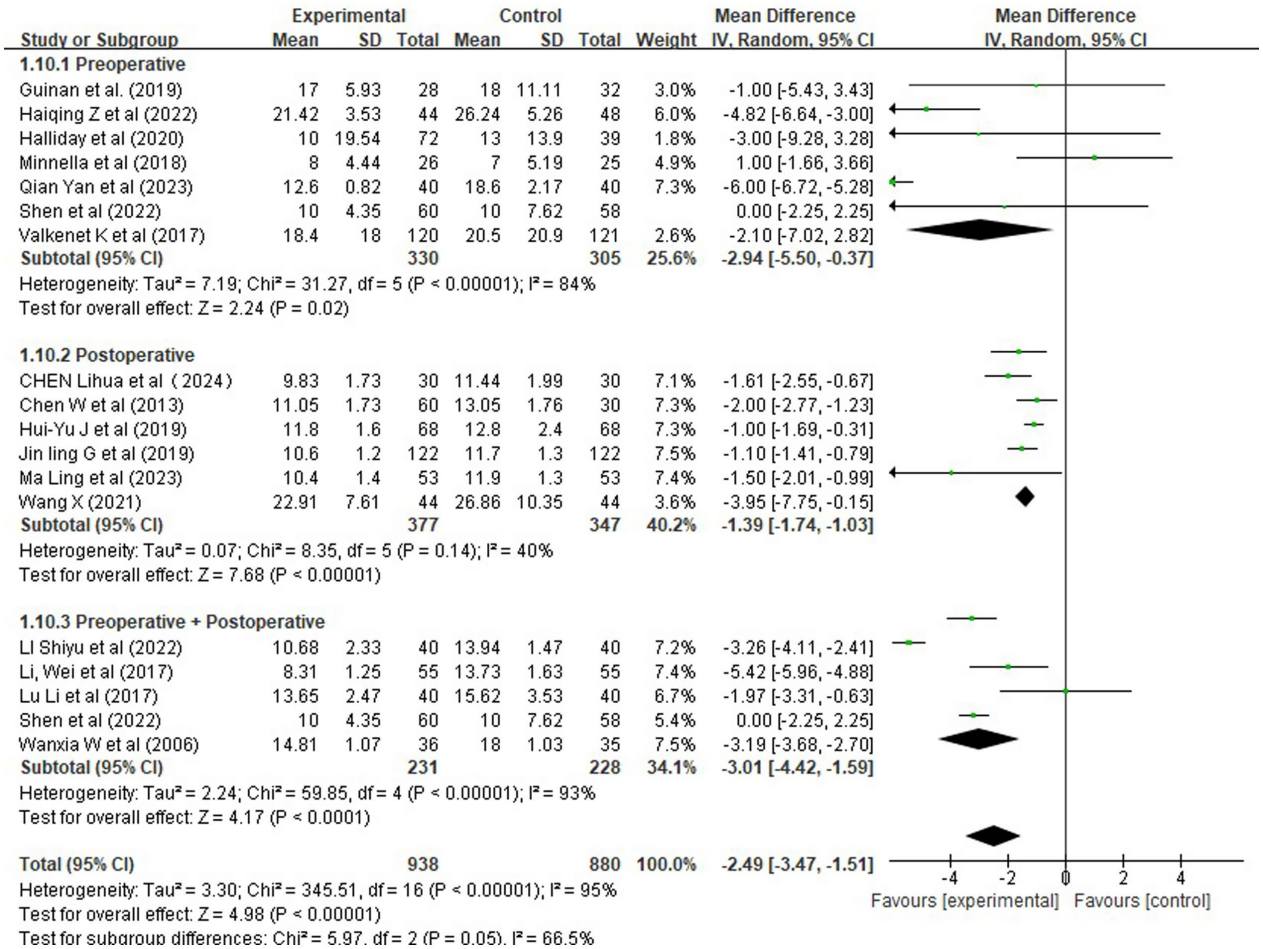
Forest plots of rehabilitation effect on length of hospital stay by intervention timing.

To explore potential sources of heterogeneity, a subgroup analysis was conducted based on the timing of rehabilitation intervention (Figure 11). The results indicated that:

Preoperative-only rehabilitation reduced hospital stay by −2.94 days [95% CI (−5.50, −0.37)], with moderate heterogeneity (*I^2^* = 84%) and borderline significance (Z = 2.24, *p* = 0.02);

Postoperative-only rehabilitation demonstrated a significant benefit [MD = −1.39 days, 95% CI (−1.74, −1.03), *p* < 0.00001], with low heterogeneity (*I^2^* = 40%);

Comprehensive perioperative rehabilitation yielded the largest reduction [MD = −3.01 days, 95% CI (−4.42, −1.59), *p* < 0.0001], with high heterogeneity (*I^2^* = 93%).

Given the extreme heterogeneity (*I^2^* = 95%), we performed a sensitivity analysis to evaluate whether the overall effect was unduly influenced by methodologically weak or ambiguously reported studies. Four studies were excluded based on pre-specified criteria: total sample size <20, lack of clear intervention description, or high risk of bias in randomization or outcome assessment. Re-analysis of the remaining 14 high-quality RCTs yielded a mean reduction in hospital stay of −2.09 days [95% CI (−2.99, −1.18), *p* < 0.00001], with heterogeneity persisting at a similarly high level (*I^2^* = 94%). Although the point estimate was slightly attenuated compared to the main analysis (MD = −2.49), the effect remained clinically meaningful and highly significant. This suggests that the association between rehabilitation and shorter hospital stays is robust across well-conducted trials, even though the magnitude varies widely.

These findings indicate that structured rehabilitation programs—particularly those initiated before surgery—are associated with reduced hospitalization duration, which may reflect improved clinical efficiency and patient flow. However, due to high heterogeneity, caution is warranted in generalizing these results to all settings. Future research should aim to identify optimal components and timing to standardize benefits.

#### 3.4.5 Quality of life

The effects of prehabilitation and rehabilitation on health-related quality of life (HRQoL) were assessed using data from nine studies involving a total of 737 patients. These studies utilized various validated instruments, including the European Organization for Research and Treatment of Cancer Quality of Life Questionnaire Core 30 (EORTC QLQ-C30) and the Short Form-36 Health Survey (SF-36). Given the heterogeneity in measurement tools, the standardized mean difference (SMD) was used as the effect size metric.

**Figure 12:**
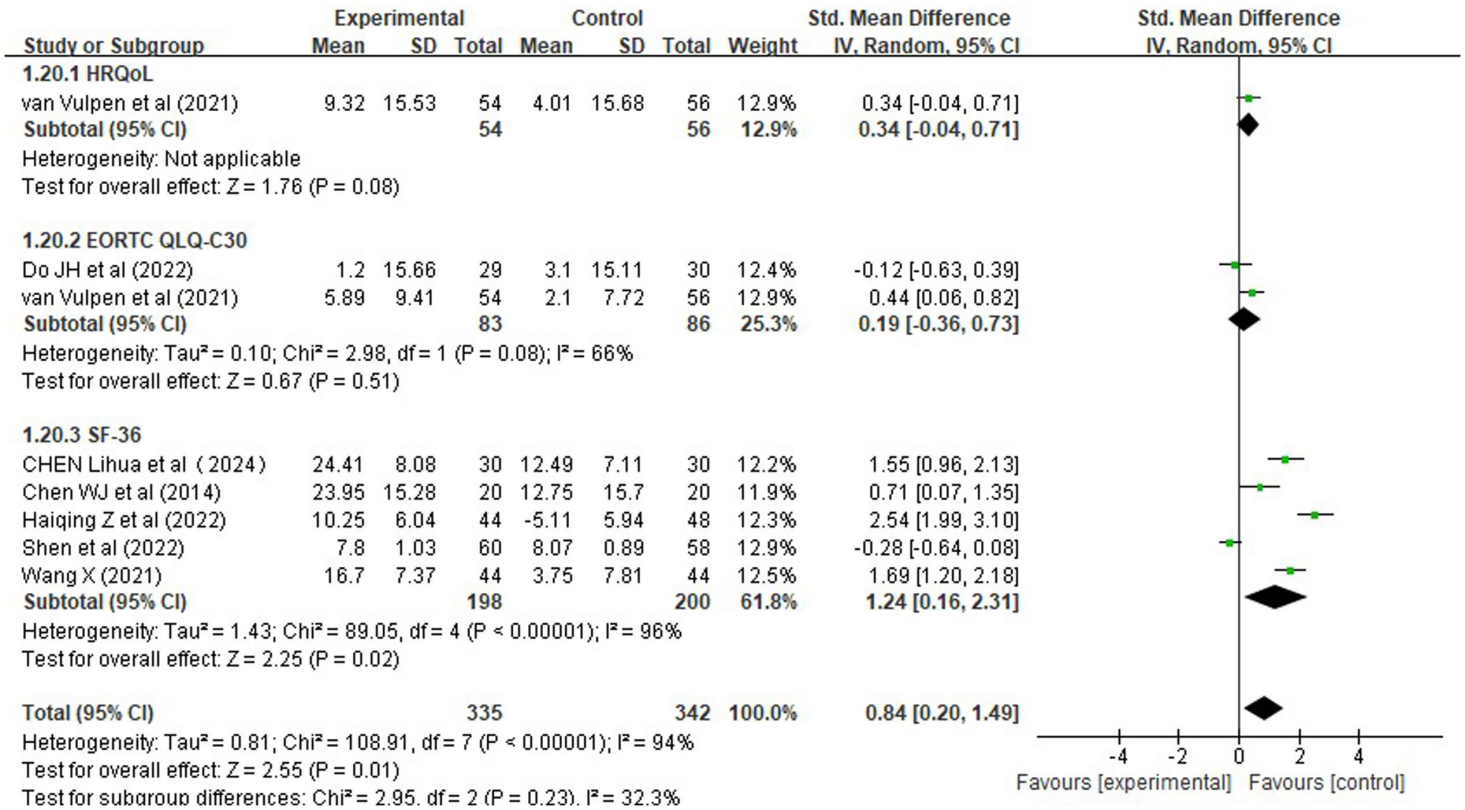
Forest plots of rehabilitation effect on health-related quality of life by assessment instrument. EORTC, European Organisation for Research and Treatment of Cancer; SD, standard deviation; IV, inverse variance; CI, confidence interval; df, degrees of freedom; QoL, quality of life.

Meta-analysis results (Figure 12) demonstrated that interventions combining preoperative and postoperative components were associated with a statistically significant improvement in HRQoL: SMD = 0.84, 95% CI [0.20, 1.49], Z = 2.55, *p* = 0.01. There was low to moderate heterogeneity among the different measurement instruments (*χ^2^* = 2.95, df = 2, *p* = 0.23; *I^2^* = 32.3%), suggesting consistent but not identical effects across scales.

Subgroup Analysis by Measurement Instrument:

- *EORTC QLQ-C30*: The pooled effect size was small and not statistically significant: SMD = 0.19, 95% CI [−0.36, 0.73], *p* = 0.51, indicating no clear benefit in cancer-specific domains.
- *SF-36*: Analysis of SF-36 scores showed a highly significant improvement in HRQoL among patients in the intervention group: SMD = 1.24, 95% CI [0.16, 2.31], *p* = 0.02, supporting beneficial effects on general physical and mental health components.

In summary, these findings indicate that prehabilitation and rehabilitation interventions are associated with significant improvements in HRQoL, particularly when assessed using the SF-36 scale. The overall effect is large in magnitude and statistically robust, but its interpretation should be tempered by the fact that it is primarily driven by one instrument. The lack of benefit in cancer-specific domains suggests that rehabilitation may enhance general well-being without necessarily improving disease-related symptoms. These results support the integration of rehabilitation into standard perioperative care pathways to improve patient-reported outcomes, especially in terms of general health status.

## 4 Discussion

This systematic review and meta-analysis suggest that perioperative rehabilitation, when aligned with the principles of Enhanced Recovery After Surgery (ERAS), may offer modest but clinically meaningful benefits for patients undergoing esophageal cancer surgery. Specifically, comprehensive perioperative rehabilitation (prehabilitation plus postoperative rehabilitation) was associated with a significantly reduced incidence of postoperative pneumonia and improved functional exercise capacity (as measured by the 6-min walk test). In contrast, prehabilitation alone demonstrated the greatest reduction in hospital length of stay (MD = −2.94 days) and significantly improved cardiopulmonary function (FEV₁, SMD = 0.60). Health-related quality of life was enhanced across multimodal programs. However, these findings must be interpreted in light of substantial heterogeneity in intervention design—ranging from isolated breathing exercises to comprehensive multimodal protocols—and high statistical inconsistency across several outcomes. These observations align with, yet extend, the findings of Tukanova et al., (51), whose recent systematic review highlighted the general benefit of physiotherapy after esophagectomy but did not differentiate the effects of prehabilitation, postoperative rehabilitation, or their integration.

Among the 37 included studies, respiratory exercises (e.g., diaphragmatic breathing, pursed-lip breathing), aerobic training, and resistance exercises were commonly employed across prehabilitation and postoperative rehabilitation programs. Respiratory training was the most frequently reported component—particularly in prehabilitation protocols—reflecting the high risk of pulmonary complications following esophagectomy, a procedure that often compromises diaphragmatic integrity and inspiratory muscle function. Such exercises aim to enhance airway clearance, promote alveolar recruitment, and improve ventilatory efficiency.

Notably, however, protocols varied widely in duration (5–15 min per session), frequency (2–3 sessions/day), and delivery method. Some studies utilized respiratory trainers (e.g., threshold inspiratory muscle devices) to increase inspiratory muscle endurance and reduce fatigue (14, 20, 26). Compliance with these devices may be limited by accessibility, cost, or patient motivation, particularly during the preoperative phase when patients may underestimate their surgical risk (28). These inconsistencies underscore the urgent need for standardized, evidence-based exercise prescriptions tailored to each phase of perioperative care.

### 4.1 Exercise intensity and safety

In addition, analysis of the literature revealed that only three of the 37 included studies described exercise intensity. Zhou Haiqing et al. (29, 30) used target heart rate [(220 − age) × 70%–80%] and maintained Borg scores at 4–6. Chen Wujing et al. (31) individualized duration based on RPE (12, 13, 19, 25). One study (30) defined clear stopping criteria: exercise was discontinued when patients reported a Borg score ≥13, experienced obvious dyspnea, had oxygen saturation <88%, or exhibited other signs of discomfort. The near-universal lack of intensity monitoring limits reproducibility and raises safety concerns, especially in frail older adults. Future trials should standardize intensity parameters and incorporate real-time physiological feedback to ensure both safety and therapeutic efficacy.

### 4.2 Advantages of multi-stage integrated intervention

Our analysis demonstrates that the timing and integration of rehabilitation components are critical determinants of therapeutic efficacy. Regarding the prevention of postoperative pneumonia—one of the most common and serious complications following esophagectomy—only comprehensive perioperative rehabilitation (i.e., combined prehabilitation and postoperative rehabilitation) was associated with a statistically significant and highly consistent reduction in risk: RR = 0.34, 95% CI [0.19, 0.61]; *p* < 0.0001, with no heterogeneity (*I^2^* = 0%). In contrast, neither prehabilitation alone [RR = 0.80, 95% CI (0.52, 1.22)] nor postoperative rehabilitation alone [RR = 1.00, 95% CI (0.65, 1.53)] showed a significant protective effect. This pattern strongly suggests that synergy between pre- and postoperative phases is essential for effective pulmonary protection, rather than either phase acting in isolation.

To explore whether surgical approach modified this benefit, we conducted a subgroup analysis among the 14 studies reporting procedure type [9 minimally invasive esophagectomy (MIE), 5 open]. The pooled effect across all studies remained non-significant overall [RR = 0.78, 95% CI (0.58, 1.06), *p* = 0.11], but notable differences emerged by technique. Specifically, comprehensive rehabilitation showed a strong trend toward reduced pneumonia risk in open surgery [RR = 0.66, 95% CI (0.40, 1.07), *p* = 0.09], whereas no discernible effect was observed in MIE patients [RR = 0.96, 95% CI (0.64, 1.44), *p* = 0.85]. Although the interaction test was non-significant (*χ^2^* = 1.39, *df* = 1, *p* = 0.24), suggesting insufficient evidence to claim differential effects, the magnitude of benefit appeared substantially greater in the higher-risk open cohort. This pattern aligns with clinical expectations: given the greater diaphragmatic disruption and systemic inflammatory response associated with open esophagectomy, structured perioperative rehabilitation may offer greater marginal utility in this setting. Conversely, the inherently lower complication profile of MIE may limit the detectable added value of rehabilitation, particularly in smaller trials with limited statistical power.

For other outcomes, however, prehabilitation alone proved highly effective: it achieved the greatest reduction in hospital length of stay (LOS), with a mean difference (MD) of −2.94 days, closely followed by comprehensive perioperative rehabilitation (MD = −3.01 days). This indicates that early physiological optimization plays a central role in accelerating discharge readiness. Conversely, postoperative-only rehabilitation did not demonstrate a significant effect on pneumonia prevention, lung function, or functional capacity in our analysis; its potential contribution to recovery under high-intensity delivery warrants further investigation but cannot be confirmed from current evidence.

The robustness of these findings was supported by sensitivity analyses. Excluding four studies judged at high risk of bias, with unclear intervention descriptions, sample sizes < 20, or lacking a control group did not materially alter the core conclusions: the significant protective effect of comprehensive perioperative rehabilitation against postoperative pneumonia persisted, and beneficial effects on hospital length of stay and 6-min walk distance remained directionally consistent, though attenuated for resource-use outcomes. Nevertheless, substantial heterogeneity persisted across functional and resource-use outcomes (*I^2^* = 91%–95%), likely reflecting variability in intervention design, intensity, and reporting.

These nuanced results challenge the assumption that “more phases always yield better outcomes.” Instead, they support an outcome-tailored model: while pneumonia prevention appears to require integrated care spanning both pre and postoperative periods, improvements in hospital stay and baseline physiological reserve may be primarily achievable through preoperative preparation.

Notably, the term “rehabilitation” was used inconsistently across included studies. Most protocols were unimodal—typically limited to breathing exercises or light mobilization—whereas only a minority adhered to Enhanced Recovery After Surgery (ERAS) principles by integrating three or more core domains, such as structured physical training, nutritional optimization, and patient education. This conceptual and operational dilution likely contributes substantially to the high heterogeneity observed in key outcomes, including hospital length of stay (*I^2^* = 95%) and 6-min walk distance (*I^2^* = 91%).

Given that postoperative pneumonia independently increases 30-day mortality by 3 to 5-fold and drives substantial healthcare costs, the 66% relative risk reduction (RR = 0.34) associated with comprehensive perioperative rehabilitation may contribute to meaningful improvements in short-term outcomes and represents a clinically important preventive strategy. Critically, this benefit was observed even in the higher-risk setting of open esophagectomy—where advanced surgical technologies may be unavailable—suggesting that structured, multi-phase rehabilitation could, in principle, serve as a scalable and equitable strategy where core components can be reliably delivered, even in resource-constrained settings.

### 4.3 Comparison of rehabilitation models

Internationally, structured perioperative rehabilitation—spanning diagnosis through post-discharge—is increasingly embedded within extended care models. For example, Loughney et al. (32) implemented a personalized program guided by the FITT principle (Frequency, Intensity, Time, Type), delivered via center-based or home-based formats with remote support over 6 weeks post-discharge. In contrast, rehabilitation in China remains largely confined to the inpatient period (typically ≤2 weeks post-surgery), often limited to postoperative exercises without preoperative preparation. Given our findings, expanding services to the preoperative phase may represent a potentially high-yield and cost-effective opportunity, particularly in settings with adequate infrastructure and patient engagement capacity.

To bridge this gap, we propose a phased transition toward a continuous, patient-centered rehabilitation ecosystem in China:

- Establish a community rehabilitation network: Drawing on foreign experience, build a community rehabilitation service system that covers a wide range of areas, so that patients can continue to receive professional rehabilitation guidance and support after they are discharged from the hospital.
- Formulate personalized rehabilitation plan: according to the specific conditions of patients, formulate personalized rehabilitation training plan, covering the whole process of preoperative preparation, intraoperative management and postoperative recovery, to ensure the continuity and effectiveness of rehabilitation training.
- Strengthening patient education and compliance management: improving patient compliance and ensuring the quality and effectiveness of rehabilitation training through the production of instructional videos and the provision of one-on-one support calls.
- Promoting the application of remote monitoring technology: utilizing modern information technology means, such as remote monitoring and m-health applications, to realize real-time tracking and feedback of the patient’s recovery process and improve the efficiency of recovery.

Overall, our findings align with ERAS guidelines but refine their implementation: not all rehabilitation is equally effective. The greatest benefits emerge from tailored strategies—comprehensive perioperative rehabilitation for pneumonia prevention, and prehabilitation for optimizing baseline physiology and reducing resource use. Given the current predominance of simplified, postoperative-only protocols in clinical practice, the full potential of perioperative rehabilitation remains unrealized.

### 4.4 Strength of evidence and implications for practice

In response to concerns that the perceived benefits of “multimodal rehabilitation” may be inferential rather than empirically substantiated, we conducted a formal GRADE (Grading of Recommendations Assessment, Development and Evaluation) assessment for all key outcomes (Supplementary Table S4). This revealed that only the reduction in postoperative pneumonia with comprehensive perioperative rehabilitation was supported by moderate-certainty evidence (⊕ ⊕ ⊕○), justifying its clinical relevance. In contrast, benefits for length of hospital stay, 6-min walk distance, FEV₁, and health-related quality of life were rated as low to very low certainty (⊕ ⊕ ○○ to ⊕○○○), primarily due to extreme heterogeneity (*I^2^* = 91%–97%) and inconsistent intervention definitions. Importantly, most included studies delivered unimodal interventions (e.g., breathing exercises alone); none directly compared standardized multimodal versus unimodal protocols. Thus, while integrated care appears beneficial for pneumonia prevention, the presumed superiority of “multimodal” rehabilitation for other outcomes remains hypothesis-generating rather than statistically confirmed.

Importantly, the clinical interpretability of pooled estimates for hospital length of stay (*I^2^* = 95%) and functional capacity (*I^2^* = 86%) is substantially limited by extreme statistical heterogeneity. While sensitivity analyses confirm that the direction of effect is consistent across higher-quality trials, the wide dispersion of true effects suggests that the observed mean differences—such as a 2.94-day reduction in LOS—should not be viewed as a universal expectation. Rather, these averages likely represent the net effect of highly context-dependent interventions, where success depends on specific combinations of exercise modality, intensity, delivery personnel, and patient characteristics. Without individual participant data or standardized reporting of intervention components (e.g., via TIDieR checklist), it remains impossible to identify which elements drive efficacy. Consequently, while perioperative rehabilitation shows promise, current evidence does not support a one-size-fits-all recommendation for reducing hospital stay or improving function; instead, it underscores the urgent need for pragmatic trials that test defined, scalable protocols in diverse healthcare settings.

### 4.5 Limitations

This study has several limitations that should be acknowledged.

First, substantial heterogeneity across included studies—in terms of intervention content, outcome definitions, and study designs—led to high statistical inconsistency in key outcomes, particularly length of hospital stay (*I^2^* = 95%) and 6-min walk distance (*I^2^* = 91%). Although random-effects models were used, such extreme heterogeneity may limit the reliability of pooled estimates. This issue is especially pronounced in subgroup analyses with few studies; for instance, the finding that comprehensive perioperative rehabilitation reduced pneumonia risk was based on only five trials, yielding limited statistical power to confirm the differential effects of intervention timing.

Second, most interventions were simplified or unimodal (e.g., breathing exercises alone) and lacked standardized reporting of exercise intensity, adherence, or integration of core ERAS components such as nutritional support or patient education. As a result, we could not evaluate the added value of truly multimodal, guideline-concordant rehabilitation programs. Moreover, despite the inclusion of resistance training in some protocols—a key strategy for preserving muscle mass in cancer surgery—few studies reported objective measures of muscle strength or body composition, hindering mechanistic insights into functional recovery.

Third, the evidence base remains constrained by a limited number of high-quality randomized controlled trials and short follow-up periods (typically ≤3 months), which precludes assessment of long-term impacts on survival, functional independence, or healthcare utilization. Critical clinical variables—such as surgical approach (open vs. minimally invasive), neoadjuvant therapy status, and baseline pulmonary function—were inconsistently reported, limiting our ability to explore effect modification in clinically relevant subgroups.

Finally, insufficient data on intervention dose (e.g., session duration, frequency, progression) prevented meta-regression analyses to examine dose–response relationships. Small-study effects may also have influenced subjective outcomes like quality of life. To advance implementation in real-world settings, future studies should adopt core outcome sets, use standardized intervention reporting (e.g., TIDieR checklist), and consider sharing individual participant data to support more precise, scalable, and equitable rehabilitation strategies—particularly in resource-variable healthcare systems.

## 5 Conclusion

This meta-analysis suggests that perioperative rehabilitation aligned with Enhanced Recovery After Surgery (ERAS) principles is associated with clinically relevant benefits for patients undergoing esophagectomy, although the certainty of evidence—graded as low to moderate by GRADE—varies considerably across outcomes.

Comprehensive perioperative rehabilitation (integrating prehabilitation and postoperative components) was the only approach significantly associated with a large reduction in postoperative pneumonia risk (RR = 0.34), supporting the potential value of continuous pulmonary support throughout the surgical trajectory. In contrast, prehabilitation alone was associated with the greatest observed reduction in hospital length of stay (MD = −2.94 days) and showed significant improvement in FEV₁, suggesting its role in enhancing preoperative physiological reserve. Multimodal programs generally improved functional capacity and health-related quality of life, though estimates were limited by substantial heterogeneity (*I^2^* > 85%).

Despite these promising signals, the current evidence base is constrained by methodological variability, frequent use of unimodal interventions (e.g., isolated breathing exercises), and limited implementation of truly integrated, ERAS-aligned multimodal rehabilitation. To advance clinical practice, future efforts should prioritize:

- Standardization of phase-specific protocols: Develop consensus-based guidelines defining core components, intensity, and delivery modalities for prehabilitation and postoperative rehabilitation, using validated outcome measures to improve comparability.
- Context-adapted implementation in China: Given the current emphasis on short-term, in-hospital postoperative care, China could pilot integrated models that extend rehabilitation into the preoperative period via community health centers or digital platforms (e.g., tele-rehabilitation), thereby harnessing the potential of prehabilitation to reduce resource use and accelerate recovery.

Moving forward, the focus should shift from simply “providing rehabilitation” to delivering the right intervention, at the right time, for the right outcome—a precision approach that may help realize the full potential of perioperative care to improve function and quality of life in esophageal cancer surgery.

## Data Availability

The original contributions presented in the study are included in the article/Supplementary material, further inquiries can be directed to the corresponding author.
