# Supplementary material for: Effects of different rehabilitation strategies on physical function and complications in postoperative patients with esophageal cancer: a systematic review and meta-analysis
Source: Front Public Health. 2026 Apr 14;14:1788265. doi: 10.3389/fpubh.2026.1788265 (PMC13121318; doi:10.3389/fpubh.2026.1788265)
Supplement: Supplementary file 1 [file Data_sheet_1.docx]

**SUPPLEMENTAL TABLES**

Supplementary table S1: search strategy

| **Database** | **Query** | **Items found** |
| --- | --- | --- |
| PubMed | #1  ("Preoperative Exercise"[Mesh]) OR "Rehabilitation"[Mesh]) OR "Exercise"[Mesh]) OR "Respiration"[Mesh]) OR "Breathing Exercises"[Mesh]  #2  (kinesiology[Title/Abstract]) OR (TCM rehabilitation[Title/Abstract]) OR (Accelerated postoperative recovery[Title/Abstract]) OR (Eight paragraphs of brocade[Title/Abstract]) OR (Home pulmonary rehabilitation[Title/Abstract]) OR (Comprehensive pulmonary rehabilitation[Title/Abstract]) OR (Resistance movement[Title/Abstract]) OR (Comprehensive respiratory function training[Title/Abstract]) OR (Active breathing training[Title/Abstract]) OR (Active breathing circuit training[Title/Abstract]) OR (Cardiopulmonary rehabilitation[Title/Abstract]) OR (inspiratory muscle training[Title/Abstract]) OR (Respiratory exercise[Title/Abstract]) OR (Blow the balloon[Title/Abstract]) OR (ERAS[Title/Abstract]) OR (Early movement[Title/Abstract]) OR (Lung/Pulmonary rehabilitat*[Title/Abstract]) OR (enhanced postsurgical recovery/ERAS[Title/Abstract]) OR (fast-track[Title/Abstract]) OR (enhanced recovery protocol[Title/Abstract]) OR (qigong[Title/Abstract]) OR (walk[Title/Abstract]) OR (strength[Title/Abstract]) OR (cycl*[Title/Abstract]) OR (Wuqinxi[Title/Abstract]) OR (respir*[Title/Abstract])  #3  "Esophageal Neoplasms"[Mesh]  #4  (chronic disease[Title/Abstract]) OR (Esophagectomy/methods[Title/Abstract]) OR (Esophageal surgery*[Title/Abstract]) OR (Esophageal tumor[Title/Abstract]) OR (esophageal cancer[Title/Abstract]) OR (Radical resection of esophageal cancer[Title/Abstract])  #5  (effect[Title/Abstract]) OR (questionnaire[Title/Abstract]) OR (scale[Title/Abstract]) OR (Evaluation study[Title/Abstract])  ((#1OR#2) AND (#3OR#4)) AND (#5) | 2472 |
| Embase | #1  'esophagus tumor'/exp OR 'esophagus tumor'  #2  'chronic disease':ti,kw,ab OR 'esophagectomy/methods':ti,kw,ab OR 'esophageal surgery*':ti,kw,ab OR 'esophageal tumor':ti,kw,ab OR 'esophageal cancer':ti,kw,ab OR 'radical resection of esophageal cancer':ti,kw,ab  #3  'exercise'/exp OR 'enhanced recovery after surgery'/exp OR 'rehabilitation'/exp  #4  'kinesiology':ti,kw,ab OR 'tcm rehabilitation':ti,kw,ab OR 'accelerated postoperative recovery':ti,kw,ab OR 'eight paragraphs of brocade':ti,kw,ab OR 'home pulmonary rehabilitation':ti,kw,ab OR 'comprehensive pulmonary rehabilitation':ti,kw,ab OR 'resistance movement':ti,kw,ab OR 'comprehensive respiratory function training':ti,kw,ab OR 'active breathing training':ti,kw,ab OR 'active breathing circuit training':ti,kw,ab OR 'cardiopulmonary rehabilitation':ti,kw,ab OR 'inspiratory muscle training':ti,kw,ab OR 'respiratory exercise':ti,kw,ab OR 'blow the balloon':ti,kw,ab OR 'eras':ti,kw,ab OR 'early movement':ti,kw,ab OR 'lung/pulmonary rehabilitat*':ti,kw,ab OR 'enhanced postsurgical recovery/eras':ti,kw,ab OR 'fast-track':ti,kw,ab OR 'enhanced recovery protocol':ti,kw,ab OR 'qigong':ti,kw,ab OR 'walk':ti,kw,ab OR 'strength':ti,kw,ab OR 'cycl*':ti,kw,ab OR 'wuqinxi':ti,kw,ab OR 'respir*':ti,kw,ab  #5  'effect':ti,kw,ab OR 'questionnaire':ti,kw,ab OR 'scale':ti,kw,ab OR 'evaluation study':ti,kw,ab  #6:#1 OR #2  #7: #3 OR #4  #5 AND #6 AND #7 | 4728 |
| Web of Science | #1  TS = (“Esophageal Neoplasms” or “chronic disease” or “Esophagectomy/methods” or “Esophageal surgery*” or “Esophageal tumor” or “esophageal cancer” or “Radical resection of esophageal cancer”)  #2  TS=(“ exercise” or “ enhanced recovery after surgery” or “ rehabilitation” or “ Breathing training” or “ Breath*” or “ kinesiology” or “ TCM rehabilitation” or “ Accelerated postoperative recovery” or “ Eight paragraphs of brocade” or “ Home pulmonary rehabilitation” or “ Comprehensive pulmonary rehabilitation” or “ Resistance movement” or “ Comprehensive respiratory function training” or “ Active breathing training” or “ Active breathing circuit training” or “ Cardiopulmonary rehabilitation” or “ inspiratory muscle training” or “ Respiratory exercise” or “ Blow the balloon” or “ ERAS” or “ Early movement” or “ Lung/Pulmonary rehabilitat*” or “ enhanced postsurgical recovery/ERAS” or “ fast-track” or “ enhanced recovery protocol” or “ qigong” or “ walk” or “ strength” or “ cycl*” or “ Wuqinxi” or “ respir*” )  #3  TS=(“effect” or “questionnaire” or “ scale” or “ Evaluation study” )  #1 AND #2 AND #3 | 2700 |
| Cochrane | #1  MeSH descriptor: [Physical Therapy Modalities] explode all trees OR  MeSH descriptor: [Rehabilitation] explode all trees OR  MeSH descriptor: [Physical Therapy Specialty] explode all trees OR  MeSH descriptor: [Rehabilitation Nursing] explode all trees OR  (physiotherapy OR mobilisation OR mobilization OR physical therap* OR physical treatment OR rehabilitat* OR exercis* OR kinesi*therap* OR incentive spiromet* OR respiratory muscle training OR breathing technique* OR inspiratory muscle training OR respiratory therap* OR positive pressure expiration OR positive expiratory pressure OR positive pressure breathing OR expansion breathing OR expansion exercise* OR cough* exercise OR ventilatory muscle training):ti,ab,kw  #2  MeSH descriptor: [Esophagectomy] explode all trees OR  MeSH descriptor: [Esophageal Neoplasms] explode all trees OR  (oesophagectomy OR esophagectomy OR esophagus resection OR oesophagus resection OR esophageal resection OR oesophageal resection OR ivor lewis OR mckeown):ti,ab,kw OR  ((oesophagus OR esophagus OR oesophageal OR esophageal):ti,ab,kw AND (cancer OR carcinoma OR neoplasm* OR tumo*r OR malign* OR adenocarcino* OR squamous):ti,ab,kw)  #3  #1 AND #2  #3  Limit #3 to yr=”1990-20241031” | 1079 |
| CINAHL | #1  physiotherapy OR physical therap* OR mobili?ation OR physical treatment OR rehabilitat* OR physical activit* OR exercis* OR kinesi*therap* OR incentive spiromet* OR respiratory muscle* training OR inspiratory muscle* training OR respiratory therap* OR breathing technique* OR positive expiratory pressure OR positive pressure expiration OR positive pressure breathing OR exercise recovery OR kinesiotherapy OR kinesitherapy OR muscle* exercise* OR expansion breathing OR expansion exercise* OR cough* exercise* OR ventilatory muscle* training OR (MH “Activities of Daily Living+”) OR (MH "Early Ambulation") OR (MH "Home Rehabilitation+") OR (MH "Physical Therapy+") OR (MH "Rehabilitation, Pulmonary+") OR (MH "Chest Physical Therapy+") OR (MH "Therapeutic Exercise+") OR (MH "Respiratory Therapy+") OR (MH "Recovery, Exercise")  #2  (oesophagectomy OR esophagectomy OR ivor lewis OR mckeown) OR [(esophagus OR oesophagus OR esophageal OR oesophageal) AND (cancer OR tumor OR tumour OR carcinoma OR neoplasm* OR malign*)] OR (MH "Esophageal Neoplasms") OR (TI "Esophagectomy / methods" OR AB "Esophagectomy / methods") OR (TI "Esophageal surgery*" OR AB "Esophageal surgery*") OR (TI "Esophageal tumor" OR AB "Esophageal tumor") OR (TI "esophageal cancer" OR AB "esophageal cancer") OR (TI "Radical resection of esophageal cancer" OR AB "Radical resection of esophageal cancer")  #3  #1 AND #2  #4  #3 AND “Published Date: 19900101-20241031” | 897 |
| CNKI | (TKA=食管癌+食管肿瘤) AND (TKA=呼吸+吹气球+咳嗽+咳痰+排痰+激励式肺活量计+呼吸肌+吸气肌+呼气肌+肺康复+有氧+抗阻+运动+锻炼+活动+训练+功率自行车+骑车+步行+慢走+瑜伽+八段锦+气功+五禽戏 +楼梯) AND (TKA=术后+手术+肿瘤切除) | 1302 |
| 中国生物医学文献数据库 | ((((((((("食管癌"[标题:智能] OR "食管肿瘤"[标题:智能])) AND (("呼吸"[标题:智能] OR [标题:智能] OR "吹气球"[标题:智能] OR [标题:智能] OR "咳嗽"[标题:智能] OR "咳痰"[标题:智能] OR "排痰"[标题:智能] OR "激励式肺活量计"[标题:智能] OR "呼吸肌"[标题:智能] OR "吸气肌"[标题:智能] OR "呼气肌"[标题:智能] OR "肺康复"[标题:智能] OR "有氧"[标题:智能] OR "抗阻"[标题:智能] OR "运动"[标题:智能] OR "锻炼"[标题:智能] OR "活动"[标题:智能] OR "训练"[标题:智能] OR "功率自行车"[标题:智能] OR "骑车"[标题:智能] OR "步行"[标题:智能] OR "慢走"[标题:智能] OR "瑜伽"[标题:智能] OR "八段锦"[标题:智能] OR "气功"[标题:智能] OR "五禽戏"[标题:智能] OR "楼梯"[标题:智能])))) AND (("术后"[标题:智能] OR "手术"[标题:智能] OR "肿瘤切除"[标题:智能])))))) AND (("效果"[标题:智能] OR "评价"[标题:智能] OR "量表"[标题:智能] OR "结局"[标题:智能]))) | 43 |
| 万方 | 主题:(食管癌 or 食管肿瘤) and 主题:(呼吸 or 吹气球 or 咳嗽 or 咳痰 or 排痰 or 激励式肺活量计 or 呼吸肌 or 吸气肌 or 呼气肌 or 肺康复 or 有氧 or 抗阻 or 运动 or 锻炼 or 活动 or 训练 or 功率自行车 or 骑车 or 步行 or 慢走 or 瑜伽 or 八段锦 or 气功 or 五禽戏 or 楼梯) and 主题:(术后 or 手术 or 肿瘤切除) | 1600 |

Supplemental table S2: PRISMA 2020 checklist

| **Section and Topic** | **Item #** | **Checklist item** | **Location where item is reported** |
| --- | --- | --- | --- |
| **TITLE** | | |  |
| Title | 1 | Identify the report as a systematic review. | P1 |
| **ABSTRACT** | | |  |
| Abstract | 2 | See the PRISMA 2020 for Abstracts checklist. | Abstract |
| **INTRODUCTION** | | |  |
| Rationale | 3 | Describe the rationale for the review in the context of existing knowledge. | P2 |
| Objectives | 4 | Provide an explicit statement of the objective(s) or question(s) the review addresses. | P2 |
| **METHODS** | | |  |
| Eligibility criteria | 5 | Specify the inclusion and exclusion criteria for the review and how studies were grouped for the syntheses. | P3 |
| Information sources | 6 | Specify all databases, registers, websites, organisations, reference lists and other sources searched or consulted to identify studies. Specify the date when each source was last searched or consulted. | P3 |
| Search strategy | 7 | Present the full search strategies for all databases, registers and websites, including any filters and limits used. | P3, suppl. table 1 |
| Selection process | 8 | Specify the methods used to decide whether a study met the inclusion criteria of the review, including how many reviewers screened each record and each report retrieved, whether they worked independently, and if applicable, details of automation tools used in the process. | P3 |
| Data collection process | 9 | Specify the methods used to collect data from reports, including how many reviewers collected data from each report, whether they worked independently, any processes for obtaining or confirming data from study investigators, and if applicable, details of automation tools used in the process. | P3-4 |
| Data items | 10a | List and define all outcomes for which data were sought. Specify whether all results that were compatible with each outcome domain in each study were sought (e.g. for all measures, time points, analyses), and if not, the methods used to decide which results to collect. | P3 |
|  | 10b | List and define all other variables for which data were sought (e.g. participant and intervention characteristics, funding sources). Describe any assumptions made about any missing or unclear information. | P3 |
| Study risk of bias assessment | 11 | Specify the methods used to assess risk of bias in the included studies, including details of the tool(s) used, how many reviewers assessed each study and whether they worked independently, and if applicable, details of automation tools used in the process. | P4 |
| Effect measures | 12 | Specify for each outcome the effect measure(s) (e.g. risk ratio, mean difference) used in the synthesis or presentation of results. | P3 |
| Synthesis methods | 13a | Describe the processes used to decide which studies were eligible for each synthesis (e.g. tabulating the study intervention characteristics and comparing against the planned groups for each synthesis (item #5)). | P4-5 |
|  | 13b | Describe any methods required to prepare the data for presentation or synthesis, such as handling of missing summary statistics, or data conversions. | P4 |
|  | 13c | Describe any methods used to tabulate or visually display results of individual studies and syntheses. | P3-4 |
|  | 13d | Describe any methods used to synthesize results and provide a rationale for the choice(s). If meta-analysis was performed, describe the model(s), method(s) to identify the presence and extent of statistical heterogeneity, and software package(s) used. | P3-4 |
|  | 13e | Describe any methods used to explore possible causes of heterogeneity among study results (e.g. subgroup analysis, meta-regression). | P4 |
|  | 13f | Describe any sensitivity analyses conducted to assess robustness of the synthesized results. | P4 |
| Reporting bias assessment | 14 | Describe any methods used to assess risk of bias due to missing results in a synthesis (arising from reporting biases). | P4 |
| Certainty assessment | 15 | Describe any methods used to assess certainty (or confidence) in the body of evidence for an outcome. | N/A |
| **RESULTS** | | |  |
| Study selection | 16a | Describe the results of the search and selection process, from the number of records identified in the search to the number of studies included in the review, ideally using a flow diagram. | P5, figure 1 |
|  | 16b | Cite studies that might appear to meet the inclusion criteria, but which were excluded, and explain why they were excluded. | P5, figure 1 |
| Study characteristics | 17 | Cite each included study and present its characteristics. | P5-9 Table 1, Table 2 |
| Risk of bias in studies | 18 | Present assessments of risk of bias for each included study. | P11, figure 2 |
| Results of individual studies | 19 | For all outcomes, present, for each study: (a) summary statistics for each group (where appropriate) and (b) an effect estimate and its precision (e.g. confidence/credible interval), ideally using structured tables or plots. | P5, Table 1 |
| Results of syntheses | 20a | For each synthesis, briefly summarise the characteristics and risk of bias among contributing studies. | P9 |
|  | 20b | Present results of all statistical syntheses conducted. If meta-analysis was done, present for each the summary estimate and its precision (e.g. confidence/credible interval) and measures of statistical heterogeneity. If comparing groups, describe the direction of the effect. | P9-18 |
|  | 20c | Present results of all investigations of possible causes of heterogeneity among study results. | P9-18 |
|  | 20d | Present results of all sensitivity analyses conducted to assess the robustness of the synthesized results. | N/A |
| Reporting biases | 21 | Present assessments of risk of bias due to missing results (arising from reporting biases) for each synthesis assessed. | P9 |
| Certainty of evidence | 22 | Present assessments of certainty (or confidence) in the body of evidence for each outcome assessed. | N/A |
| **DISCUSSION** | | |  |
| Discussion | 23a | Provide a general interpretation of the results in the context of other evidence. | P18-21 |
|  | 23b | Discuss any limitations of the evidence included in the review. | P20, 21 |
|  | 23c | Discuss any limitations of the review processes used. | P20, 21 |
|  | 23d | Discuss implications of the results for practice, policy, and future research. | P21 |
| **OTHER INFORMATION** | | |  |
| Registration and protocol | 24a | Provide registration information for the review, including register name and registration number, or state that the review was not registered. | P2-3 |
|  | 24b | Indicate where the review protocol can be accessed, or state that a protocol was not prepared. | P2-3 |
|  | 24c | Describe and explain any amendments to information provided at registration or in the protocol. | N/A |
| Support | 25 | Describe sources of financial or non-financial support for the review, and the role of the funders or sponsors in the review. | P21 |
| Competing interests | 26 | Declare any competing interests of review authors. | P22 |
| Availability of data, code and other materials | 27 | Report which of the following are publicly available and where they can be found: template data collection forms; data extracted from included studies; data used for all analyses; analytic code; any other materials used in the review. | Suppl. material |

**Sensitivity Table S3. Summary of Main and Sensitivity Analyses for Key Outcomes**

| **Outcome** | **Analysis** | **Studies (n)** | **Effect (SMD)** | **95% CI** | **I² (%)** | **p-value** |
| --- | --- | --- | --- | --- | --- | --- |
| 6MWD | Main | 9 | 0.89 | [0.39, 1.38] | 84 | <0.0001 |
|  | Sensitivity* | 7 | 0.94 | [0.43, 1.45] | 82 | 0.0004 |
| Length of Hospital Stay | Main | 17 | –2.49 | [–3.47, –1.51] | 95 | <0.0001 |
|  | Sensitivity* | 14 | –2.09 | [–2.99, –1.18] | 94 | <0.0001 |
| Pneumonia Incidence | Main | 14 | 0.70 | [0.52, 0.96] | 42 | 0.02 |
|  | Sensitivity* | 10 | 0.71 | [0.53, 0.95] | 41 | 0.02 |

*Excluded studies with small sample size (<20), unclear intervention description, or high risk of bias.

**Supplementary Table S4. GRADE Evidence Profile for Key Outcomes**

| **Outcome** | **No. of studies / Participants** | **Effect estimate [95% CI]** | **Evidence Certainty** | **Net Benefit** | **Risk/Burden** | **Patient Values** | **Feasibility** | **Reasoning** |
| --- | --- | --- | --- | --- | --- | --- | --- | --- |
| Postoperative pneumonia incidence (All rehabilitation vs control) | 16 RCTs / 1,627 patients | RR 0.70 [0.52, 0.96] | ⊕⊕⊕○ | High | Low | High | High | Consistent benefit with moderate heterogeneity; low-risk, high-impact intervention. |
| Length of hospital stay (All rehabilitation vs control) | 14 RCTs / 1,818 patients | MD –2.49 days [–3.47, –1.51] | ⊕⊕○○ | Moderate | Low | Moderate | High | Significant reduction but very high heterogeneity limits generalizability. |
| 6-minute walk distance (All rehabilitation vs control) | 9 RCTs / 572 patients | SMD 0.90 [0.41, 1.38] | ⊕⊕○○ | Moderate | Low | Moderate | Moderate | Large effect but high heterogeneity due to variable protocols. |
| FEV₁ (All rehabilitation vs control) | 9 RCTs / 721 patients | SMD 0.60 [0.31, 0.89] | ⊕⊕○○ | Uncertain | Low | Low–Moderate | Moderate | Modest improvement; clinical relevance unclear. |
| Health-related quality of life (HRQoL) (All rehabilitation vs control) | 8 RCTs /677 patients | SMD 0.84 [0.20, 1.49] | ⊕○○○ | Moderate | Low | High | Moderate | Large effect but extreme heterogeneity and inconsistent measurement tools. |

**Notes**:

- Certainty levels: High (⊕⊕⊕⊕), Moderate (⊕⊕⊕○), Low (⊕⊕○○), Very Low (⊕○○○).
- Net benefit considers magnitude and consistency of effect; all interventions carried low risk.
- Feasibility: "High" = easily scalable; "Moderate" = requires staff training or equipment.
- CI = confidence interval; MD = mean difference; RR = risk ratio; SMD = standardized mean difference.

**Funnel Plots Assessing Publication Bias Across Key Outcomes: (1) 6-Minute Walk Test, (2) Cardiopulmonary Function, (3) Incidence of Pneumonia, (4) Length of Hospital Stay, and (5) Health-Related Quality of Life**


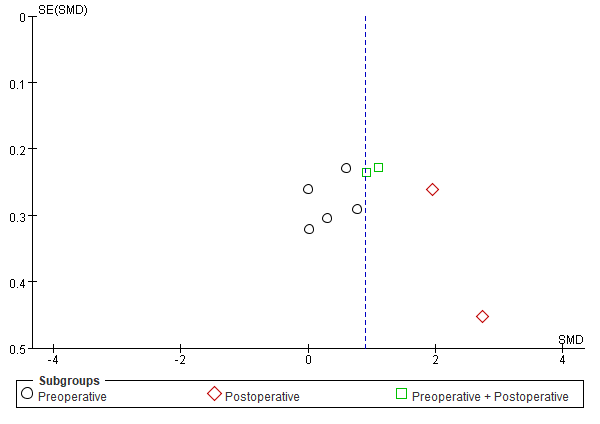
**
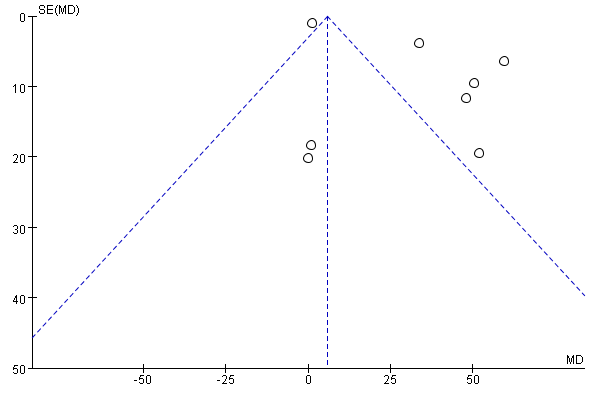

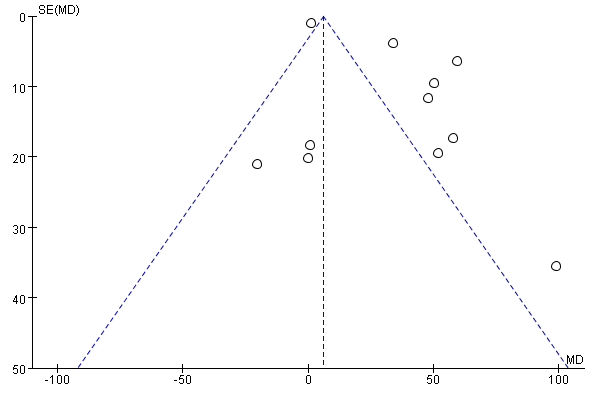
**（1）6-Minute Walk Test

a. All studies b. Includes only RCTs c. Different timing of intervention

**
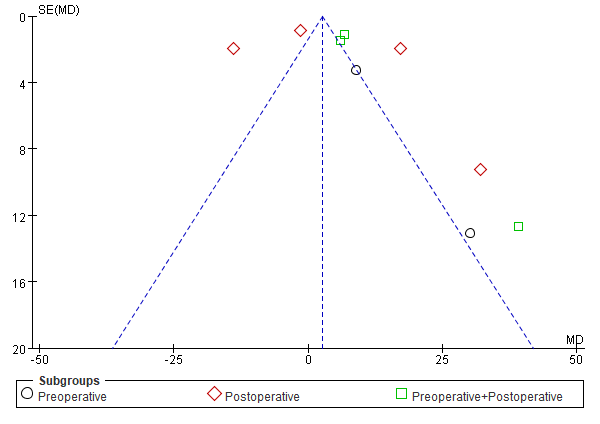

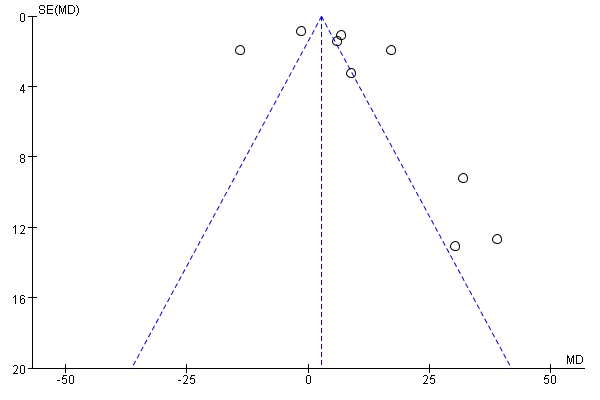
**(2) Cardiopulmonary Function Test

c. All studies d. Different timing of intervention

**
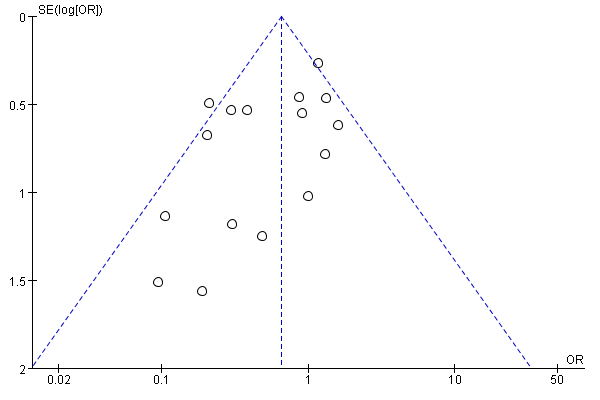
**(3) Incidence of Pneumonia

**
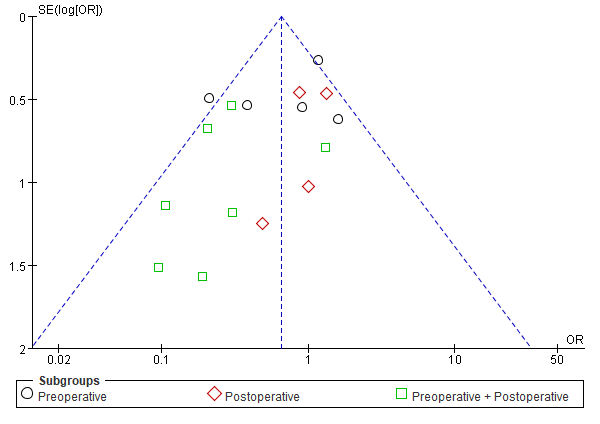

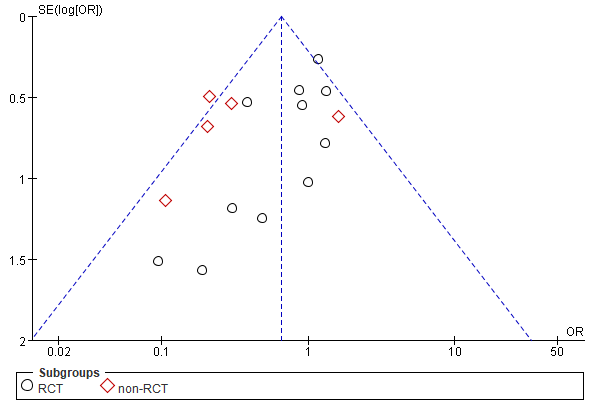
**

e. All studies f. RCTs and non-RCTs g. Different timing of intervention

(4) Length of Hospital Stay


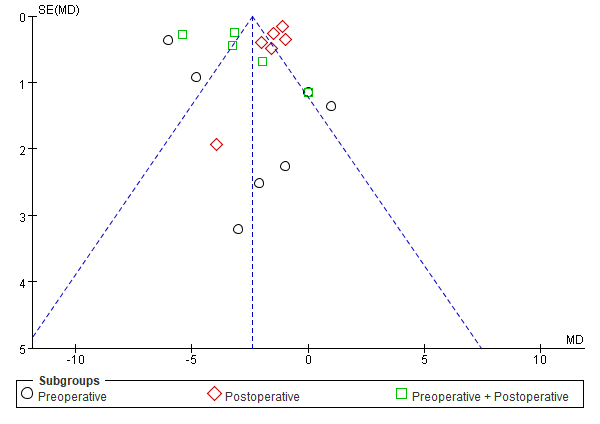

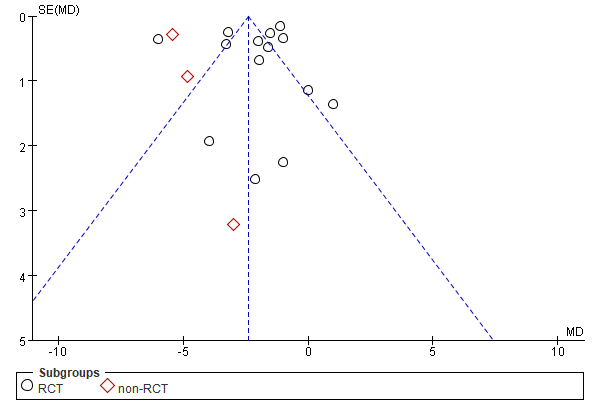

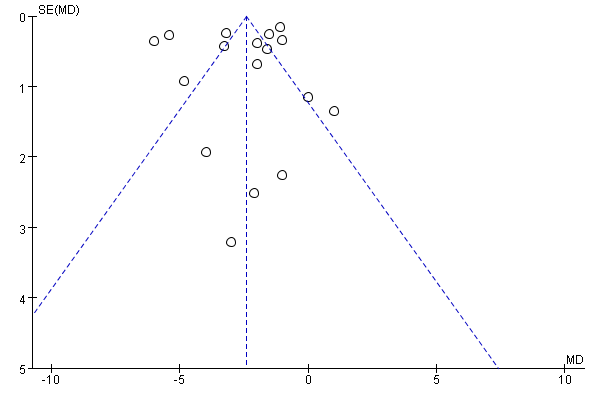


h. All studies i. RCTs and non-RCTs j. Different timing of intervention

*Note: Due to small sample sizes in some subgroups (e.g., HRQoL, n=5), funnel plots should be interpreted with caution. No significant asymmetry was observed across most outcomes.*
